# From pedagogical toward technological pedagogical content knowledge frameworks and their effectiveness in teaching mathematics: A mapping review

**DOI:** 10.12688/f1000research.125073.1

**Published:** 2022-09-12

**Authors:** Hashituky Telesphore Habiyaremye, Celestin Ntivuguruzwa, Philothere Ntawiha

**Affiliations:** 1 African Center of Excellence for Innovative Teaching and Learning of Mathematics and Science (ACEITLMS), University of Rwanda College of Education (URCE), Kayonza, Rwanda; 2 School of Education, University of Rwanda College of Education (URCE), Kayonza, Rwanda

**Keywords:** Google Scholar, framework, mathematics, pedagogical content knowledge, technological pedagogical and content knowledge

## Abstract

Background: A study to reveal existing pedagogical or technological pedagogical content knowledge frameworks is crucial to inform and their effectiveness in teaching mathematics. This review study intended to explore the trends of the pedagogical content knowledge (PCK) framework, how it has changed over time until the most recent version of technological and pedagogical content knowledge (TPACK) was developed, and its effectiveness in teaching mathematics.

Methods: We initially downloaded 273 articles from the first 30 Google Scholar pages and analyzed 229 journal articles. We got 24 frameworks from 64 journal articles since Shulman’s first model in 1986. About 52 out of 229 were mathematics studies. Among these studies, we found that 18 studies have extensively investigated the use of identified frameworks.

Results: The frameworks were presented and descriptively discussed in chronological order. The empirical studies that compared the role of pedagogical and technological pedagogical content knowledge models among classrooms with teachers who possess and do not possess such skills were demonstrated.

Conclusions: The gap in empirical studies was identified, and further studies about the intervention of PCK and TPACK models were suggested to gain more insight into the mathematics classroom.

## Introduction

Around the 1980s, a new era in subject matter and teacher pedagogy rose. Shulman (1986) argues that the emphasis on teacher material knowledge and pedagogy is conserved as special. He believes that teacher training should conglomerate these two areas of specialization. He introduced the concept of pedagogical content knowledge (PCK), which comprises pedagogical knowledge (PK) and content knowledge (CK), to address this dichotomy, amongst other classifications. His original portrayal of the teacher's knowledge encompassed information about the curriculum and knowledge of the educational context.

After two decades, a major revolution has been proposed by various researchers in the field of technology. Mishara and Koehler (2006) established the concept of technological pedagogical and content knowledge (TPACK) in reaction to the lack of theory guiding technology integration into teaching. TPACK characterizes an extension of Shulman’s (1986) representation of what their colleagues looked for to teach explicit content (that is, PCK) by depicting the knowledge required to teach such content with technology ( Mishara & Koehler, 2006).

### Problem statement

The old review article we found was written in 2008 and discussed how English teachers across institutional networks develop PCK ( Smith & Anagnostopoulos, 2008), while the latest review was published in 2020 from Njiku *et al.* (2020) and analyzes research instruments used and authenticated by 28 diverse studies to measure teacher TPACK.

Among 20 review articles we found, 15 were journal articles, two were conference proceedings, two were book chapters, and one was a book. Among these 15 journal articles, none investigated accumulating PCK framework over the years, or gave a clear analysis into teaching intervention through those frameworks, and teachers’ difficulties in attaining content knowledge. For instance, the current methods and instruments of TPACK were reviewed by Abbitt (2011) in the training of pre-service teachers, Aydin (2012) researched the field of science teacher training in the framework of the PCK in Turkey, and the validity and reliability of using TPACK as a reference framework for determining the extent to which teachers can teach using technology was done by Young *et al.* (2012). In 2013, Chai *et al.* reviewed 74 journal articles exploring information and communication technology (ICT) integration from the TPACK system, an investigation of the theoretical foundation and the functional use of TPACK ( Voogt *et al.*, 2013) was done, Depaepe *et al.* (2013) systematically reviewed PCK in the way the concept permeated mathematics pedagogical research, and Wu (2013) reviewed empirical TPACK studies that were published in prominent and international journals from 2002 to 2011.

One article gave a critical review of PCK’s nature, principles, and trends ( HU, 2014), another gave an analysis of TPACK studies in Turkey using a meta-synthesis method and showed the types of trends in this field ( Yilmaz, 2015), and another by Rosenberg and Koehler (2015) provided an inclusive and truthful picture of the degree to which background is encompassed in the TPACK. Evens *et al.* (2016) reviewed PCK in connection with teaching foreign and second languages; Hubbard (2018) summarized how PCK computing is conceptualized and investigated in computer pedagogical research. Rossi and Trevisan (2018) investigated how TPCK is defined and affected in the early career teaching profession. Grieser and Hendricks (2018) prepared a literature review on PCK, which is specific to the topic and to the music education environment in general to better understand problems that teachers with limited knowledge of string specific content may experience when teaching string students. Therefore, our study aimed at reviewing:

1. Chronological trends in PCK models (from beginning to date, how did researchers modify the original model that Shulman started with? Which models, what was modified, why were the modifications made, which teachers and/or students benefited from that change?)
2. Effectiveness of PCK and TPACK frameworks (empirical studies that have used these frameworks to upgrade students learning. Any level, primary-secondary-university. Any year. Descriptive and inferential statistics each study got, etc.).

## Methodology

### Data selection

**Figure 1.**
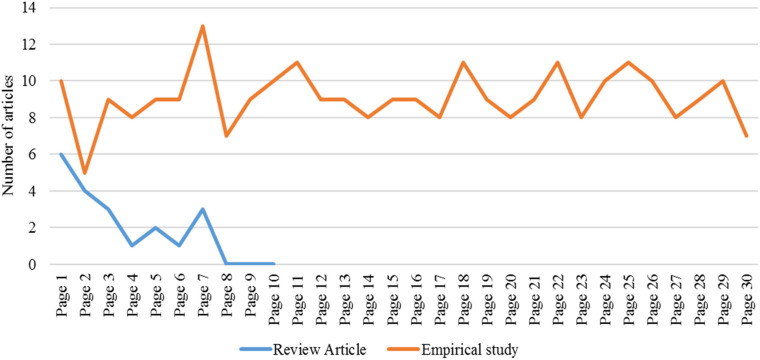
Number of studies downloaded from each page of Google Scholar.

We designed and employed a “one-term in one-source” method. This method involves using one term, a key, or search word in only one academic search engine. Thus, we have used “pedagogical content knowledge” as the search term in Google Scholar as an academic source. This search returned 20 review papers and 273 empirical studies at a glance (see Figure 1) on 19 August 2021.

Using the term “pedagogical content knowledge” in Google Scholar, we explored the results in the first 30 pages. We downloaded every article that contains the words “pedagogical content knowledge” in its title. We only included articles written in English. The first paper, *“Pedagogical content knowledge in social studies,”* was published in 1987, while the latest *“A virtual internship for developing technological pedagogical content knowledge”* was published in 2020. The first seven pages exhausted all articles written in review form, such as a review of literature, systematic review, meta-analysis, etc. This means that we checked pages eight to 10 and had no results and stopped checking after page 10. There were many more empirical studies (articles that investigate with primary data), so we limited the search to the 30 ^th^ page (entry); as you can see, the last page still had seven articles. We did not limit ourselves to year of coverage, subject, or grades level. However, we intended to explore mathematical PCK explicitly. Thus, we retrieved all articles regardless of subject but then later excluded the non-mathematics articles. Literature review articles were visited only to frame our research problem, while empirical articles were focused on the analysis to answer our research objectives. The first author did initial screening and the co-authors checked the inclusion criteria.

### Extended sources and nature of selected articles

Although we used only one database, we found articles from other repositories. For instance, 24 articles were from the educational resources information center (ERIC), 17 from Academia, 13 from Research Gate, two from HAL ( *Hyper Articles en Ligne*), and two from Durham University. However, all these were accessed via Google Scholar. Thus, articles in Google Scholar will take you to the website they are hosted on or repository they are deposited on. About the nature of articles selected, 229 were identified as journal articles, 22 were conference proceedings, 8 were book chapters, 2 were books, and 5 were generics (articles with unclear identification). Therefore, our analysis only considered journal articles.

### Data analysis

**Figure 2.**
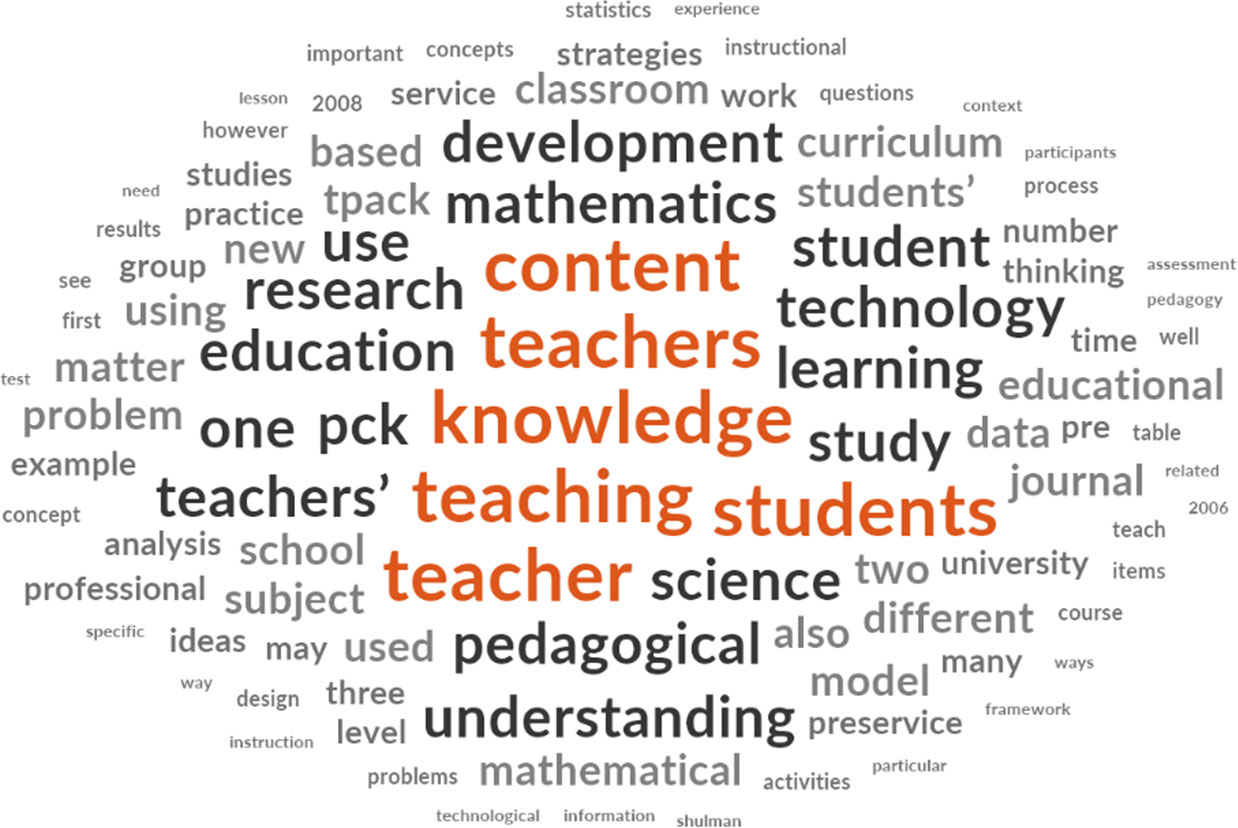
Word frequency from all downloaded articles.

PCK models were cited, presented in figures, and described. Teaching interventions were presented in sections, and their statistics were presented in tables and descriptively discussed. Teachers’ knowledge was presented descriptively or in tables by discussing the type of study (survey, cases studies, interventions, etc.) that produced the results, the type of data such as quantitative or qualitative, the type of mode such as whether teachers’ knowledge was measured from him/herself or from his/her students, etc. Theoretical strands were also described accordingly. We used both NVivo 1.0 software and MS Excel 2016 to analyze data. Figure 2 is a typical example of the word analysis from NVivo using articles that were downloaded.

**Figure 3.**
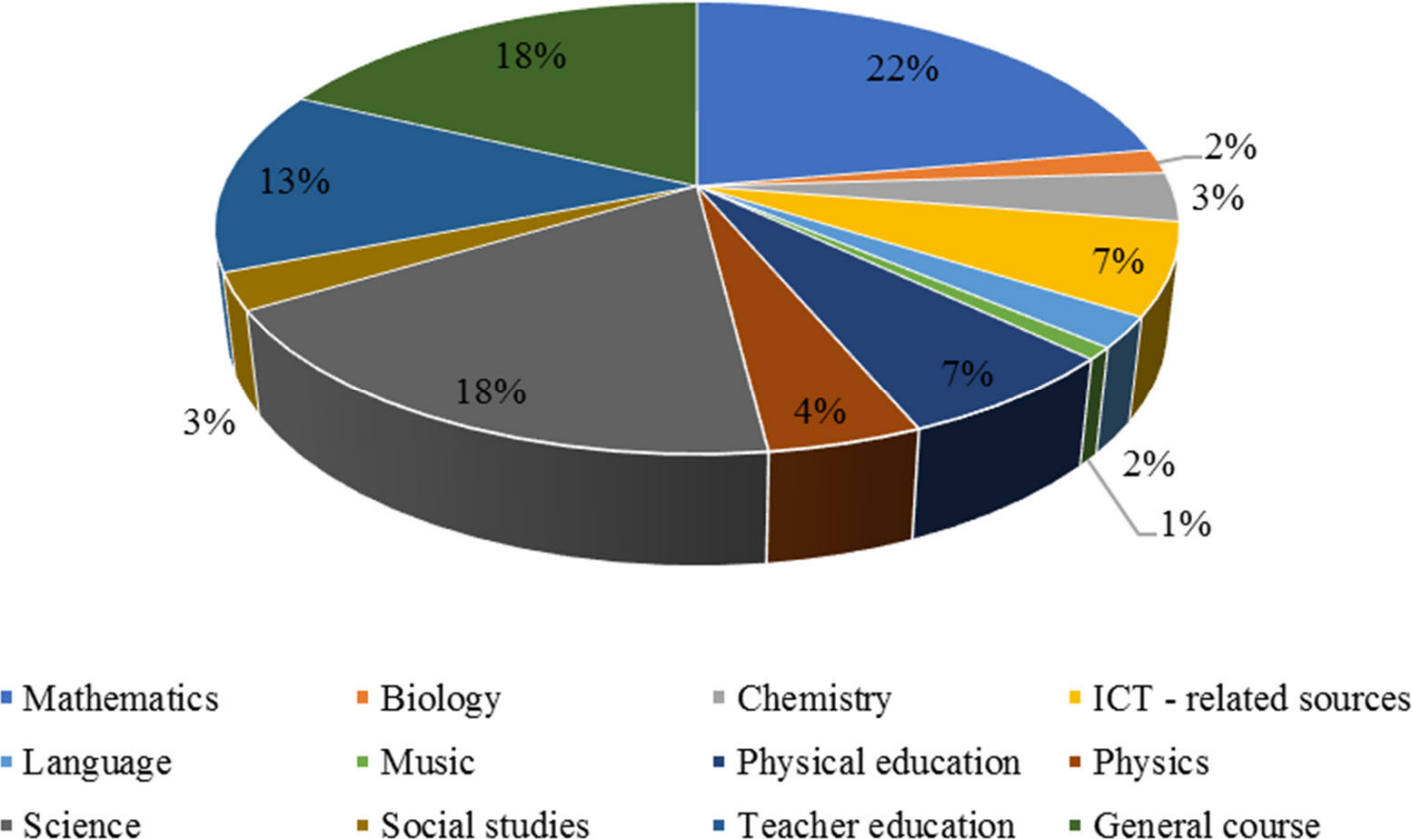
Subjects and number of articles.

12 subjects were identified across 229 articles. Articles discussing or investigating learning mathematics dominated the list (23% or 52 out of 229 articles). Science subjects and articles related to teacher education were 42 (18%) and 30 (13%) out of 229 articles, respectively, while unidentified articles or articles investigating PCK in a general sense accounted for 40 (17%). Figure 3 shows the distribution of articles among different subjects.

Among 229 retrieved journal articles, 110 (48%) were from 20 journals from various publishers. Table 1 shows that Teaching and Teacher Education, published by Elsevier, and the International Journal of Science Education, published by Taylor and Francis, were the two journals ranked first and second respectively among the top 20 contributors in this study (6% and 4%, respectively).

**Table 1.** Journals that mostly contributed to the present study.

	Journal	Publisher	Number of articles	% of articles
1	Teaching and Teacher Education	Elsevier B.V	13	6%
2	International Journal of Science Education	Tylor & Francis Ltd	10	4%
3	Computers & Education	Elsevier B.V	8	3%
4	Journal of Teacher Education	SAGE Publishing	8	3%
5	Journal of Teaching in Physical Education	Human Kinetics	8	3%
6	Australasian Journal of Educational Technology	Ascilite	6	3%
7	Journal of Research in Science Teaching	Wiley Periodicals	6	3%
8	Research in Science Education	Springer Publishing	6	3%
9	Contemporary Issues in Technology and Teacher Education	AACE	5	2%
10	International Journal of Science Education	Tylor & Francis Ltd	5	2%
11	Journal of Educational Computing Research	Baywood Publishing	5	2%
12	Journal of Science Teacher Education	Tylor & Francis Ltd	5	2%
13	Journal of Research in Science Teaching	Wiley Periodicals	4	2%
14	Educational Sciences: Theory & Practice	-	3	1%
15	Journal for Research in Mathematics Education	NCTM	3	1%
16	Journal of Digital Learning in Teacher Education	Tylor & Francis Ltd	3	1%
17	Journal of Research on Technology in Education	Tylor & Francis Ltd	3	1%
18	Research in Science & Technological Education	Tylor & Francis Ltd	3	1%
19	Research Quarterly for Exercise and Sport	Tylor & Francis Ltd	3	1%
20	Teachers and Teaching: theory and practice	Tylor & Francis Ltd	3	1%
			110	48%

AACE: Association for the Advancement of Computing in Education, NCTM: National Council of Teachers of Mathematics.

**Figure 4.**
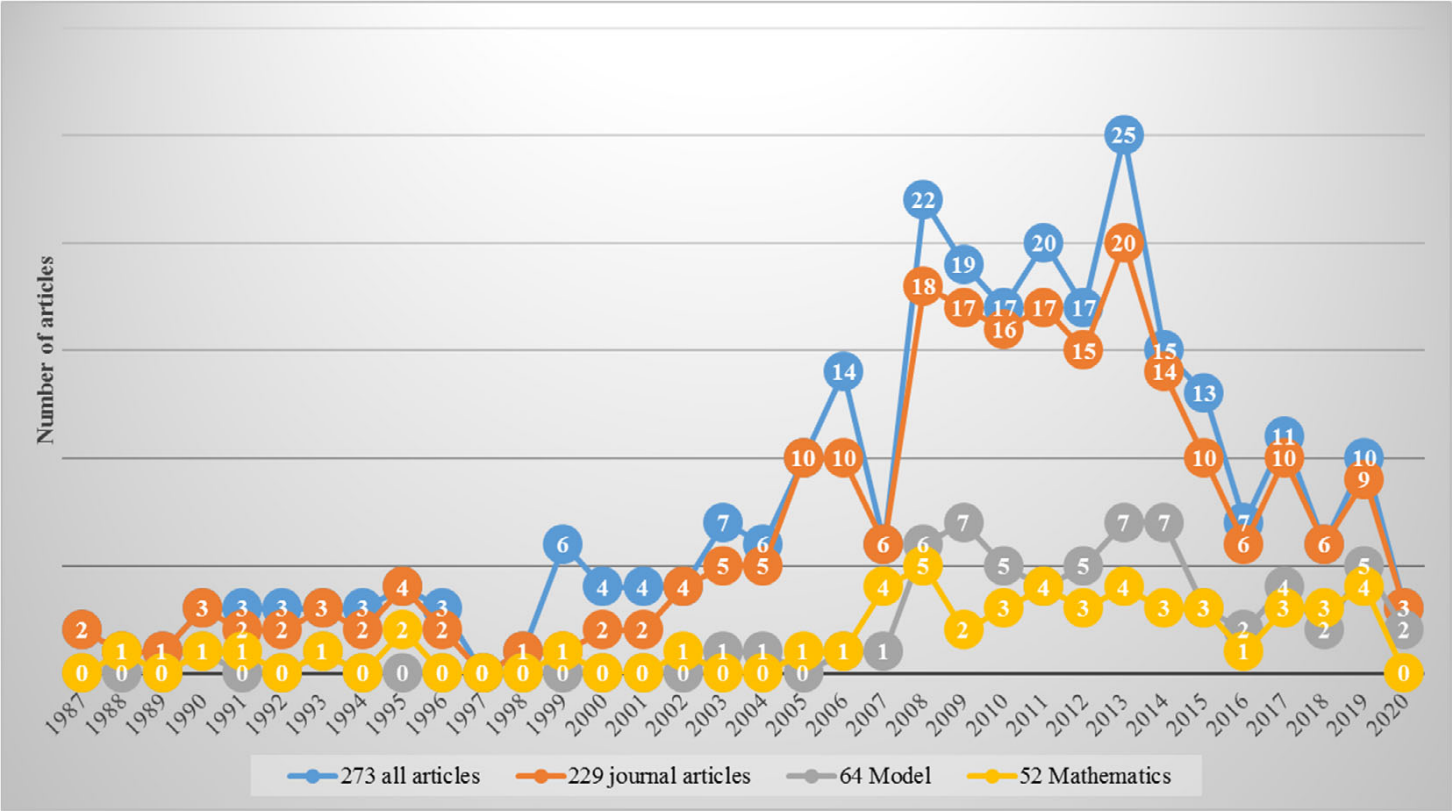
Displaying the hierarchy of analysis.

Figure 4 displays the hierarchy of analysis. The blue color chart shows the number of all empirical studies downloaded across the years of publication. The first study that investigated PCK, after its launch from Shulman in 1986, was published in 1987, while the latest came out in 2020. After filtering out proceedings, books, book chapters, theses, and generics, 229 journal articles are shown in red. After analyzing these 229 articles, we found 64 articles that clearly investigated or used PCK or TPACK frameworks or models (see green color). Thus, others investigated these models. They did not formulate new models but used existing ones. Finally, among 229 articles, 52 articles were found to extensively investigate mathematics lessons, as shown in purple.

## Results and discussion

### PCK framework

65 (including 22 related to mathematics) out of 229 showed PCK trend models. About 24 articles (including 11 related to mathematics) showed original frameworks, while 41 referred to these 24 articles. Thus, Table 2 displays a review of 24 frameworks presented in the related 24 articles. Among these 24 frameworks, 17 are versions of PCK, and seven are upgrades of TPACK.

**Table 2.** Trends of pedagogical content knowledge (PCK) framework.

	Year	Authors	Model	Construct
1	1990	( Marks, 1990)	A structure for PCK in fifth-grade equivalence of fractions	PCK
2	1993	( Geddis, 1993)	Transforming subject-matter knowledge	PCK
3	1995	( Simon, 1995)	Mathematics Teaching Cycle	PCK
4	2003	( Jones & Moreland, 2003)	7 component of framework	PCK
5	2004	( An *et al.*, 2004)	The network of PCK	PCK
6	2005	( Niess, 2005)	Four different aspects that comprise teachers’ TPCK	TPACK
7	2006	( Major & Palmer, 2006)	The PCK change process	PCK
8	2006	( Mishara & Koehler, 2006)	Pedagogical Technological Content Knowledge	TPACK
9	2008	( Hill *et al.*, 2008)	Domain map for mathematical knowledge for teaching	PCK
10	2008	( Miranda, 2008)	Infusion of Engineering Knowledge, Pedagogy and Context in Technology Education Instruction	TPACK
11	2009	( Kaya, 2009)	Components of the PSTs’ PCK investigated	PCK
12	2009	( Koehler & Mishra, 2009)	The TPACK framework and its knowledge components	TPACK
13	2010	( Etkina, 2010)	The Structure of Physics Teacher Knowledge	PCK
14	2011	( Saeli *et al.*, 2011)	Diagram based on Grossman’s reformulation of PCK	PCK
15	2011	( Tee & Lee, 2011)	Key design considerations for creating activities and conditions to facilitate socialisation, externalisation, combination and internalisation	TPACK
16	2011	( Voss *et al.*, 2011)	Conceptualization of general pedagogical/psychological knowledge (PPK)	PCK
17	2012	( McCray & Chen, 2012)	Pedagogical content knowledge	PCK
18	2012	( Saeli *et al.*, 2012)	Scheme representing the relation between Saeli *et al.*’s (2012) study and their previous ones	PCK
19	2013	( Zepke, 2013)	Revisiting PCK	PCK
	2013	( Lannin *et al.*, 2013)	A model of PCK for teaching mathematics	PCK
20	2015	( Olfos *et al.*, 2014)	Structures of the studies of the teacher’s PCK and CK	PCK
21	2019	( Gess-Newsome *et al.*, 2019)	Project PRIME theoretical path of influence/Model of teacher professional knowledge and skill including PCK	PCK
22	2019	( Chai *et al.*, 2019)	The revised scaffolded TPACK lesson design model (R-STLDM)	TPACK
23	2020	( Gasteiger *et al.*, 2020)	Explicit and implicit knowledge of early childhood teachers	PCK
24	2020	( Andyani *et al.*, 2020)	The TPACK component and extract the structural model of the mutual relationship between its constructs	TPACK

Table 2 outlines the models and constructs for each study. We are now going to discuss each of the 24 concepts in more detail.

### A structure for pedagogical content knowledge in fifth-grade equivalence of fractions

Students’ understanding, media for instruction, and subject matter are inter-related. At the same time, these components are connected to instructional processes that contain student focus, presentation focus, and media focus ( Marks, 1990, p.5).

### Transforming subject-matter knowledge

The subject-matter content knowledge (SmCK) contributes to teachable content knowledge (TCK), while PCK comes as a supplement to remedy misconceptions (for instance, using alternative representations). See Geddis (1993, p.677).

### Mathematics teaching cycle

The author emphasized assessing students’ knowledge of mathematics and mathematical activities and representations, the teacher’s capacity to hypothesize the students’ knowledge and learning of particular content, and teacher’s capability to theorize the mathematics learning and teaching. These capabilities contribute to the learning trajectory that embeds the teacher’s learning objectives, the learning activity plan, and the hypothesis of the learning process ( Simon, 1995, p.56).

### Seven components of the framework

The author outlined and described seven constructs of PCK ( Jones & Moreland, 2003, p.82): nature and characteristics of the subject; conceptual, procedural, and practical features of the topic; knowledge of curriculum; knowledge of students (such as prerequisite knowledge, strenths and weaknesses of the student, and learning progress); special teaching and assessment practice for the subject (holistic, authentic, etc.); sympathetic of the role and place of context; and classroom environment and topic management (such as resources, hardware, and technical abilities).

### The network of pedagogical content knowledge

The network is centered on students’ learning where both content, curriculum, and knowing students’ thinking (KST) are direct contributors. The network starts from beliefs that interchangeably corroborate with PCK in the center of three components (content, teaching, and curriculum) that directly interchangeably feed into the KST. KST has four other four outcomes apart from students learning. These are (a) remediating students’ misunderstandings, (b) make mathematics learning attractive to students, (c) constructing students’ mathematics ideas, and (d) endorsing students’ discerning mathematics ( An *et al.*, 2004, p.147).

### Four different aspects that comprise teachers’ TPCK

In this model, four steps are involved in its development ( Niess, 2005): an initial idea of the meaning of what to teach an actual topic with technology; understanding of teaching strategies and presentations for teaching a specific topic with technology; awareness of students’ understanding, thinking, and education with technology; familiarity of curriculum and its resources that fit in technology in students learning.

### The PCK change process

The authors emphasized the context of change. Existing PCK contributes to institutional intervention and expression of knowledge. The institutional intervention contributes to a new PCK that produces the expression of knowledge. Thus, this institutional intervention brings the context of change ( Major & Palmer, 2006, p.626).

### Pedagogical Technological Content Knowledge

The three circles (content, pedagogy, and technology) overlap to create four more interrelated forms of knowledge ( Mishara & Koehler, 2006, p.1025). These are pedagogical content knowledge (PCK), technological content knowledge (TCK), technological pedagogical knowledge (TPK), and technological pedagogical content knowledge (TPCK).

### Domain map for mathematical knowledge for teaching

The authors unpacked subject matter knowledge from PCK ( Hill *et al.*, 2008, p.377). The PCK remained in three parts: knowledge of content and teaching (KCT), knowledge of content and students (KCS), and knowledge of curriculum (KC). On the other side, SMK comprised three components: specialized content knowledge (SCK), common content knowledge (CCK), and knowledge at the mathematical horizon (KMH).

### Infusion of Engineering Knowledge, Pedagogy and Context in Technology Education Instruction

Apart from PCK, the context of technology and engineering knowledge (ETK) was added ( Miranda, 2008, p.3). Thus, the pedagogical context in engineering and technology education arises between pedagogy knowledge (PK) and ETK, while engineering and technology content in the context of representation of knowledge of content (CK) and ETK. The final product has emerged as PCK in engineering and technology (PCKET).

### Components of the PSTs’ PCK investigated

According to the authors, PCK includes both subject matter knowledge (SMK) and pedagogical knowledge (PK), where PK ( Kaya, 2009, p.964) is composed of (a) knowledge of instructional strategies and activities (KISA), (b) knowledge of curriculum (KC), (c) knowledge of students’ learning difficulties (KSLD), and (d) knowledge of assessment (KA).

### The TPACK framework and its knowledge components

Three components (content, pedagogical, and technological) of knowledge intersect ( Koehler & Mishra, 2009, p.63). The content knowledge (CK) and pedagogical knowledge (PK) contribute to PCK. CK and technological knowledge (TK) contribute to technological content knowledge (TCK). PK and TK contribute to technological pedagogical knowledge (TPK). The whole context (intersection of PCK, TCK, and TPK) produces TPACK (technological pedagogical and content knowledge).

### The Structure of Physics Teacher Knowledge

The author described what CK, PK, and PCK involve during learning physics. CK involves knowledge of physics, thoughts, associations, and approaches to evolving novel information. PK contains information about brain development, cognitive science, collaborative learning, the classroom, and knowledge about leadership and school laws. PCK includes teaching orientation, knowledge of the physics curriculum, student ideas, effective teaching strategies, and assessment methods ( Etkina, 2010, p.2).

### Diagram based on Grossman’s reformulation of PCK

Pedagogical content knowledge (PCK) is fuelled by the reason to teach, content to teach, learning difficulties, and methods of teaching ( Saeli *et al.*, 2011, p.76).

### Key design considerations for creating activities and conditions to facilitate socialization, externalization, combination, and internalization

The authors cultured TPACK from socialization to internalization through problem-based learning ( Tee & Lee, 2011, p.91). The first process is socialization (e.g., open-ended, in-class discussion, and asynchronous online debates). The second process is designing outsourcing, such as single and collective writing exercises, model or prototyping, discourse, and reflection from the group. The third process is a combination involving designing reliable or replicated multifaceted circumstances that challenge learners to fuse several facts is done. The fourth process is internalization (e.g., common written and oral replications and ideal explanations).

### Conceptualization of general pedagogical/psychological knowledge (PPK)

The general pedagogical and psychological knowledge (PPK) is composed of two components (classroom processes and students’ heterogeneity). Classroom controlling, teaching procedures, and classroom assessment reside on the side of classroom processes, while student learning progressions and individual physiognomies are catered for by the students’ heterogeneity component ( Voss *et al.*, 2011, p.954).

### Pedagogical content knowledge (PCK)

PCK was divided into three components: subject matter understanding, knowledge of students’ development, and teaching techniques ( McCray & Chen, 2012, p.295).

### Scheme representing the relation between Saeli et al.’s (2012) study and their previous ones

The terms used are Pedagogical Content Knowledge (PCK), content representation (CoRe), online PCK teachers’ analyzer (OTPA), and PCK textbook analyzer (PTA). CoRe portrays PCK and constructs OTPA to assess teachers while PTA assesses textbooks.

### Revisiting pedagogical content knowledge

The normal PCK framework was divided into two segments where the upper part was referred to as learner perspective. In contrast, the lower part was split into two and referred to as teacher designs content (CK side), and teacher designs learning environment (PK side). Teaching and learning were emphasized on the PK side ( Zepke, 2013, p.98).

### A model of PCK for teaching mathematics

PCK has four components: knowledge of the mathematics curriculum, knowledge of instructing mathematics, knowledge of assessing mathematics, and students’ comprehending within mathematics ( Lannin *et al.*, 2013, p.406).

### Structures of the studies of the teacher’s PCK and CK

Content knowledge (CK) is composed of (a) CcK (conceptual knowledge), such as conceptual understanding of fractions, language used, their uses and problem-solving; and (b) RK (representational knowledge), such as teacher illustrations of mathematical items (pictorial or figurative) about fractions. PCK is composed of (a) KTC (knowledge of the teaching of content), such as knowledge of the adaptation of mathematical facts, and (b) KSK (knowledge of students’ knowledge) ( Olfos *et al.*, 2014, p.919).

### Project PRIME theoretical path of influence

Teacher practice directly influences students’ achievement ( Gess-Newsome *et al.*, 2019, p.948). There are three components: ACK, general PK, and PCK, which are interventions for teacher practice and, therefore, student achievement.

### Model of teacher professional knowledge and skill including PCK

Teachers’ professional knowledge has five bases; curricula, content, pedagogical, assessment knowledge, and students’ knowledge. This knowledge is directly related to topic-specific professional knowledge, teacher beliefs and orientation, context, classroom practice, students’ beliefs, prior knowledge, behaviors, and finally to students outcomes ( Gess-Newsome *et al.*, 2019, p.962).

### The revised scaffolded TPACK lesson design model (R-STLDM)

The R-STLDM has two stages. The first stage is determining lesson objectives. It gathers, analyses, and diagnoses information or knowledge between instructional goals (within CK and TK) and learners’ context (within PCK and TPCK). This stage’s outcome is the design decision that connects to the second stage. The second stage connects planning of instructional and learning activities (PK and TPACK activities), the relevant choice of technologies (TPK, TCK, and TPACK resources), and assessment development (formative and summative) together ( Chai *et al.*, 2019, p.372).

### Explicit and implicit knowledge of early childhood teachers

Knowledge was divided into two components; overt and inherent. Knowledge in overt context can be activated independently from the situation, can be uttered in technical semantic, can be methodically used to plan to learn, and can be used to perceive and spot juveniles’ knowledge or abilities. On the level of implicit knowledge, it can only be triggered and expressed in situational contexts, stimulate planning of learning conditions, and lead to adequate supportive reactions to children’s performance ( Gasteiger *et al.*, 2020, p.199).

### The TPACK component and extract the structural model of the mutual relationship between its constructs

Content knowledge (CK), pedagogical knowledge (PK), and technological knowledge (TK) are drawn in a way that they contribute to TPACK. Also, PCK, TCK, and TPK contribute to TPACK. Then CK contributes to PCK and TCK, PK contributes to PCK and TPK, while TK contributes to TCK and TPK ( Andyani *et al.*, 2020, p.128).

### PCK interventions

52 out 229 were identified as articles related to mathematics. Among 52 mathematics articles, 39 were related to TPACK, while 13 were investigating PCK. 18 of the 52 articles fit our analysis framework and spanned from 1988 to 2020. They showed the implications of PCK and TPACK frameworks. Except for these 18 articles, 34 out of 52 articles reviewed showed survey results that only provided the outcome of PCK or TPACK. These 18 articles were only articles that investigated the outcome of comparative studies such as pre-and post-test designs, variables such as teachers’ backgrounds, teachers’ gender, teachers’ geographical locations, etc. Most of the studies left out were surveys without comparison, and others were qualitative. Table 3 displays a review of 18 articles related to mathematics that showed the comparative outcome of PCK and TPACK.

**Table 3.** Trends of pedagogical content knowledge (PCK) framework.

	Study	Construct	Theoretical framework	Research design	Teaching intervention	Topic	Analysis
1	Teachers’ PCK of students’ problem-solving in elementary arithmetic ( Carpenter *et al.*, 1988)	PCK	-	Survey, correlation	The study compared teachers’ estimates and students’ performance	Functions	Quantitatively descriptive
2	The Lesson Preparation Method: a way of investigating pre-service teachers’ PCK ( Van Der Valk & Broekman, 1999)	PCK	-	Survey	Lesson preparation task and the subsequent interview	Area	Qualitatively descriptive
3	Preparing teachers to teach science and mathematics with technology: Developing a technology PCK ( Niess, 2005)	TPCK	Constructivism	Pre and post-assessment	Microteaching	-	Qualitatively descriptive
4	Development of mathematics PCK in student teachers ( Lim-Teo *et al.*, 2007)	PCK	-	Pre and post-testing	Methodology course	-	Inferential statistics
5	Preparing to teach mathematics with technology: an integrated approach to developing TPACK ( Lee & Hollebrands, 2008)	TPACK	-	Quasi-experiment	Technology and an Integrated Approach	-	Inferential statistics
6	Secondary mathematics teachers’ PCK and content knowledge: validation of the COACTIV constructs ( Krauss, Baumert, *et al.*, 2008)	PCK	Theory of adult intellectual development	Teacher-students comparative study	Cognitive Activation in the Classroom (COACTIV) approach with related conceptualizations	-	Inferential statistics
7	Teaching area and perimeter: mathematics PCK in-action ( Kow & Yeo, 2008)	PCK	-	Beginning and senior teacher comparative study, classroom observation, and field notes	Lesson delivery	Area and perimeter	Qualitatively descriptive
8	PCK and content knowledge of secondary mathematics teachers ( Krauss, Brunner, *et al.*, 2008)	PCK	Generalizability theory	Correlation study	In-depth mathematical training	-	Inferential statistics
9	Mathematics teachers’ topic-specific PCK in the context of teaching a ^0^, 0! and a ÷ 0	PCK	-	Comparative study between experienced and novice teachers	-	a ^0^, 0! And ÷0	Deductive content analysis
10	From socialization to internalization: cultivating TPACK through problem-based learning ( Tee & Lee, 2011)	TPACK	-	Prior and after the course for Mathematics and Education groups	Proto-theories in the form of a problem-based learning approach guided by the socialization, externalization, combination, and internalization framework	-	Inferential statistics
11	Teacher education effectiveness: quality and equity of future primary teachers’ mathematics and mathematics PCK ( Blömeke *et al.*, 2011)	PCK	Education and social inequality	Language and gender differences in mathematics CK and mathematics PCK	-	-	Inferential statistics
12	Teachers’ content knowledge and PCK: the role of structural differences in teacher education ( Kleickmann *et al.*, 2013)	PCK	-	Cross-sectional data from four samples, pre-and in-service teachers	Cognitive Activation in the Classroom (COACTIV)	-	Inferential statistics
13	Teachers’ PCK and its relation with students’ understanding ( Olfos *et al.*, 2014)	PCK	Vergnaud’s theory of conceptual fields	An ex-post-facto non-experimental design implemented with ten variables	-	-	Inferential statistics
14	Integrating PCK and pedagogical/psychological knowledge in mathematics ( Harr *et al.*, 2014)	PCK	Bandura sub-theory on abstract modelling	Pre- and post-test design	Computer-based working memory task	-	Inferential statistics
15	Teachers’ content and PCK on rational numbers: A comparison of prospective elementary and lower secondary school teachers ( Depaepe *et al.*, 2015)	PCK	The theory of conceptual change	Pre- and in-service teachers assessed among elementary and lower secondary school teachers	Three years of professional training	Rational numbers	Inferential statistics
16	Content knowledge and PCK in Taiwanese and German mathematics teachers ( Kleickmann *et al.*, 2015)	PCK	-	Cross-sectional study and data from a sample of experienced in-service and experienced teachers	-	Algebra, Geometry	Inferential statistics
17	TPACK of mathematics teachers and the effect of demographic variables ( Ozudogru & Ozudogru, 2019)	TPACK	-	Survey design, Multivariate and Univariate analyses for gender, level of school, and teaching experience	-		Quantitatively descriptive
18	Decolonising TPACK of first-year mathematics students ( Khoza & Biyela, 2020)	TPACK	A unified theory of acceptance and use of technology	Survey and post-observation	GeoGebra resources	Algebra, Trigonometry	Qualitatively describe

Table 3 presents trends of pedagogical content knowledge framework. We are now going to discuss them in more detail and reveal their effectiveness in teaching mathematics.

### Teachers’ PCK of students’ problem-solving in elementary arithmetic

This study ( Carpenter *et al.*, 1988) examined the pedagogical content of 40 grade-one teachers about children’s explanations to word problems related to addition and subtraction in 27 schools located in Madison, Wisconsin, USA. Most teachers were able to identify many critical differences between the problems and the basic plans that children used to answer dissimilar types of issues. However, this information is often not prearranged into a cohesive linkage that connects the differences between difficulties, youths’ resolutions, and the severity of the problems. Instructors’ knowledge of whether their learners were able to solve a variety of problems correlated meaningfully with apprentice performance.

### The lesson preparation method: a way of investigating pre-service teachers’ PCK

Teacher training should be based on the knowledge base provided by trainee teachers ( Van Der Valk & Broekman, 1999). A group of math and science teachers developed a methods for studying parts of knowledge from the pedagogical content of the subject. Pre-service teachers were asked to formulate a lesson on a topic, such as an area of the body. The authors concluded that the method is useable to fully make teachers prompt their PCK.

### Preparing teachers to teach science and mathematics with technology: developing a TPCK

The development of PCK for custodial teachers was examined in terms of the integration of technology ( Niess, 2005). The four gears of the PCK have been adapted to refer to the technology boosted PCK. This research studied the TPCK of student-teachers in a multidimensional training program for science and math teachers that integrates technology teaching and learning through the software package. Five gears defined hitches and the success of technology teachers in training the TPCK. Students’ sight of incorporation, taking into account the nature of technology and science, was acknowledged as a central part of the advance of the TPCK.

### Development of mathematics PCK in student teachers

The 16 questions for the PCK of mathematics (MPCK) were established to size its aspects in the teaching of mathematics in primary school ( Lim-Teo *et al.*, 2007). The instrument was awarded to 113 of the 261 student teachers who entered the graduate program in July 2005. The MPCK was re-introduced in February 2006, shortly before teacher-students began their teaching internships. Between July 2005 and February 2006, MPCK development occurred in 96-hour computer science courses and in general education courses that include two other modules. The results show that teachers at the establishment of their education are poor in their knowledge of the pedagogical content of mathematics. There was a weighty development in all areas of the MPCK after the accomplishment of the pedagogy course.

### Preparing to teach mathematics with technology: an integrated approach to developing TPACK

Lee and Hollebrands (2008) provided examples of teacher training materials developed using an approach in which teachers' understanding of content, technology and pedagogy are prepared to teach data analysis and probability topics using specific technological tools. The fall 2005 control group (N=15) changed from 33% pre-test to 39% post-test score, spring 2006 execution of first draft of provisions (N=18) from 44% pre-test to 33% post-test score, and fall 2006 enactment after amendment for instruction (N=15) from 50% pre-test to 67% post-test score.

### Secondary mathematics teachers’ PCK and content knowledge: validation of the COACTIV constructs

In the COACTIV (Professional Competence of Teachers, Cognitively Activating Instruction, and the Development of Students’ Mathematical Literacy) project, PCK and CK tests for secondary school mathematics teachers were established and implemented with German teachers who participated in the 2003/04 Programme for International Student Assessment (PISA) longitudinal evaluation ( Krauss, Baumert, *et al.*, 2008). The second COACTIV dimension point (2004), on which the PCK and CK tests were taken, involved 218 secondary school mathematics teachers; 198 teachers completed both exams. The sample results, examined in the context of the three growing information hypotheses, appear to be a marked upsurge in acquaintance in both PCK and CK during higher education and then a smoother rise in the next phase of teacher education. Outward associations with teachers’ subjective beliefs about math and math learning show that expert teachers discard the view that math is just a toolkit and that math can superlatively be educated through cautious listening. In addition, the outcomes of structural equation modeling show that PCK supports the learning conveyed by the lesson aspects.

### Teaching area and perimeter: mathematics PCK in-action

Kow and Yeo (2008) examined the influence of knowledge of the pedagogical content of a mathematics teacher (MPCK) on the area and perimeter of teaching for four grades. Aspiring teachers’ classrooms were examined to identify the activities and teaching strategies used to identify the area and perimeter ideas. The observed results of MPCK in action are also explored through videos with a novice teacher. The complex interactions between the concepts of area and perimeter sometimes worked well for the novice teacher. At the same time, in other cases, gaps in knowledge about the content of pedagogical mathematics could mislead students.

### PCK and content knowledge of secondary mathematics teachers

Krauss, Brunner, *et al.* (2008) describe the design of tryouts to test this knowledge and the application of these tests to the models of 198 German mathematicians. They assess whether knowledge of knowledge concepts and concepts of knowledge can make a difference and whether the average knowledge and level of connection between the two knowledge groups depend on mathematical skills. Studies have shown that educators with thorough mathematics training outperformed educators from other schools in both categories of knowledge and demonstrated a large grade of cognitive connection amid the two sets of knowledge.

### Mathematics teachers’ topic-specific PCK in the context of teaching a ^0^, 0! and a ÷ 0

639 high-school students initially gave explanations about “a ^0^ = 1, 0! = 1” and “a ÷ 0” where a ≠ 0 ( Cankoy, 2010). Secondly, 58 high school mathematics teachers in Northern Cyprus wrote how they teach the abovementioned topics to high school learners. The study revealed that proficient teachers suggest more abstract teaching strategies than beginner teachers. These strategies were against PCK as they were mainly procedural, fostering memorization.

### From socialization to internalization: cultivating TPACK through problem-based learning

The results of this study recommended that a PBL-based classroom designed with an environment that supports socialization, externalization, pairing, and internalization can help teachers cultivate TPACK ( Tee & Lee, 2011). The study found that teachers were better prepared to use TPACK more productively after their cerebral models switched to Biggs’ Level 2 and Level 3 styles. The course has created chances for teachers to understand that technology alone is unlikely to advance teaching skills, and teachers can consider their teaching skills and the nature of the subject when choosing a technology.

### Teacher education effectiveness: quality and equity of future primary teachers’ mathematics and mathematics PCK

This article analyzed the extent to which primary teacher training can be considered effective and the possible causes of inequality in 15 nations ( Blömeke *et al.*, 2011). The target group for this study was demarcated as prospective teachers in the closing year of teacher education allowed to teach mathematics in basic education. Random sampling was used in a two-step process in each participating country. The study proved substantial cultural alterations in the success of teacher formation. The language was found to be divided into direct and indirect impacts. This presented an amalgamation of different options for teacher training programs rendering to teachers’ training and different achievements in these programs.

### Teachers’ content knowledge and PCK: the role of structural differences in teacher education

In a comparison study, Kleickmann *et al.* (2013) examined the CK and PCK of pre- and in-service teachers at different stages of their teaching careers. The results showed that there was a significant difference in the CKs of the teacher groups examined here. Confirmatory factor analyses revealed that reasonably invariant population reflected that PCK and CK measurement, the major variances in CK and PCK were established between the start and the close of preliminary teacher training.

### Teachers’ PCK and its relation with students’ understanding

Participants were randomly and proportionally selected by stratum in around 38% of the 144 schools in the three chief cities in Valparaíso, Chile ( Olfos *et al.*, 2014). At the beginning and end of the test, the authors used groundwork, academic, and socioeconomic formation in schools where they are grouped into the classroom. The constructivist sub-component of the authors’ framework was powerfully related with student attainment, approving the robust significance of the association with teaching experience. The CK of Socio-Economic Teachers presented an important link to learner learning, though these are divisions characteristic of the Chilean education system.

### Integrating PCK and pedagogical/psychological knowledge in mathematics

This empirical study was led in agreement with the ethical procedures of the German Psychological Society. Harr *et al.* (2014) assessed the duration of each participant’s working memory separately. In an experimental study of 60 mathematics students, authors examined the effect of giving aspects of overall pedagogical and psychological knowledge (PPK) as well as PCK in a combined or separate way. Under both experimental situations, participants were first given brief information about working with the computer program used in the treatment session. After a computer-assisted working memory task, participants worked on a previous PCK and PPK pretest. As a result, the integrated condition led to a wider application of the pedagogical and psychological features and growth in the concurrent submission of both sorts of data parallel to the separate condition. Overall, the results show the positive effects of integrated design on teacher training.

### Teachers’ content and PCK on rational numbers: A comparison of prospective elementary and lower secondary school teachers

Depaepe *et al.* (2015) researched the knowledge of potential teachers about the content (CK) and PCK in rational numbers, the connection between CK and PCK, and their dissimilarities among future teachers trained as primary and secondary school teachers. They found shortcomings in the perspective of CK and PCK teachers, a positive relationship amongst CK and PCK, and enhanced CK.

### Content knowledge and PCK in Taiwanese and German mathematics teachers

Kleickmann *et al.* (2015)’s comparison study of two nations provided additional proof on a two-dimensional edifice of the teachers’ knowledge on the subject. In addition to CK, PCK represents a special but related aspect of subject matter knowledge. The variances in CK and PCK among Taiwanese and German primary teachers after training presented in TEDS-M are more apparent than in experienced teachers who participated in this study. The teaching familiarity was not associated with the CK and PCK scores, which indicated the solidity of CK and PCK in the work stage.

### TPACK of mathematics teachers and the effect of demographic variables

This study developed and validated the TPACK scale to investigate the knowledge levels of mathematics teachers in TPACK constituents and to examine whether the TPACK stages of mathematics teachers differed ( Ozudogru & Ozudogru, 2019). Gender, teaching experience, and grade level were demographic variables. Data was collected from 202 math teachers in the spring semester of the 2016-2017 school year. As a result, there were significant transformations in gender in favor of male teachers in terms of mastery of TK. It was pointed out that teachers who teach in primary schools feel more like their TK than teachers who teach in secondary schools. While male teachers teaching in elementary school with 16 to 20 years of teaching experience rated their highest TK, female teachers in high school with 1 to 5 years of teaching experience rated their TK lowest.

### Decolonizing technological PCK of first-year mathematics students

Decolonization is a process of curriculum criticism and renewal ( Khoza & Biyela, 2020). This study suggested the submission of personal knowledge to create a practical program as an elucidation to the decolonization of education. Counting algebra is learned by students from an early age in society. In other module cases, knowledge of technology and content dominated learning, but the pedagogical knowledge that arose from introspection to understand their identity always led the module. Therefore, the study recommended that students utilize their knowledge of content, pedagogy, and technology as a taxonomy of apprenticeship to meet the needs of mathematics, individuals, and society through technology integration.

## Conclusion

**Figure 5.**
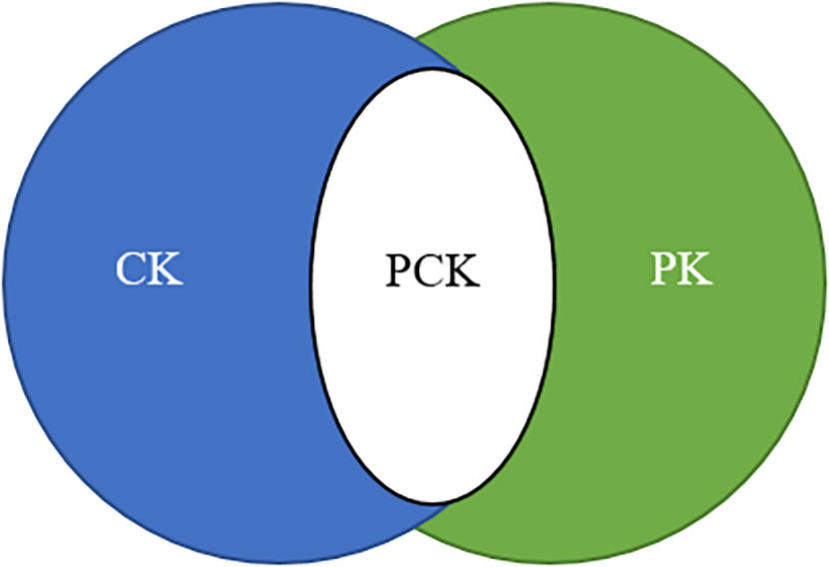
Original PCK framework, from Shulman (1986 and 1987).

Our selected method of one-term in one-source was effective to dig into literature and find the relevant information required to answer the presented research questions. In the early 1980s, Shulman (1986) initiated the idea of pedagogy as an important construct to deliver an effective lesson. In both 1986 and 1987, he demonstrated that a teacher does not only need content knowledge, but rather pedagogical knowledge too (see Figure 5), such as the knowledge of curriculum, students, and instruction ( Shulman, 1987).

Since then, other researchers have continued to develop this idea. They started to implement it in the classroom and in different contexts such as various other subjects and ICT inclusion. Different contexts have prompted researchers to modify the initial model or framework to fit their needs. This is the reason that since the early 2000s, researchers have noticed a deficiency of relevant theoretical framework in the age of technology. Therefore, various researchers (mainly Koehler & Mishra, 2005; Mishara & Koehler, 2006) designed an experiment to understand teachers’ professional development via integrating ICT in their teaching.

As a result, technological, pedagogical, content knowledge (TPCK) was offered. The term TPCK was initially used in the literature until 2008 ( Mishra & Koehler, 2008) when the academic world suggested the supplementary and easily pronounced term of technological, pedagogical, and content knowledge (TPACK). From then on, technology has been identified as an important player in the teaching and learning process as educational institutions move into the 21st century.

In this study, we opted to fill the gap in the literature of how the framework was hierarchically modified to fit effective teaching and to learn in different contexts and document their contributions to learning achievement. Therefore, we further investigated their effectiveness in refining mathematics teaching and learning by reviewing the outcomes of studies that tested PCK and TPACK frameworks. These were empirical studies that have used these frameworks as an intervention to upgrade students learning. The quick outlook showed that PCK was investigated more than TPACK – 13 out of 18 reviewed papers were related to PCK. We have found that many studies were surveying teachers on how they perform a certain component of these frameworks such as teacher subject knowledge, pedagogy, etc. Few studies focused on fostering PCK and TPACK skills to foster the students’ performance, attitude, and conceptual understanding. Thus, further studies are needed to illuminate these skills in the classroom and their outcomes on students’ learning. For instance, no study has investigated the difference between teachers with PCK skills and non-PCK skills and what the reflection of this is on their students. Future studies should also investigate the teachers’ lack of difficulties in attainment of content knowledge (CK) or pedagogical knowledge (PK) by looking into empirical studies – surveys, cases studies, interventions, etc. – that have investigated teacher knowledge (CK or PK).

## Data availability

All data underlying the results are available as part of the article and no additional source data are required.

## Author contribution

All authors have equally participated in designing and communicating the results.
