# From pedagogical content knowledge toward technological pedagogical content knowledge frameworks and their effectiveness in teaching mathematics: A mapping review

**DOI:** 10.12688/f1000research.125073.2

**Published:** 2023-08-17

**Authors:** Hashituky Telesphore Habiyaremye, Celestin Ntivuguruzwa, Philothere Ntawiha

**Affiliations:** 1 African Center of Excellence for Innovative Teaching and Learning of Mathematics and Science (ACEITLMS), University of Rwanda College of Education (URCE), Kayonza, Rwanda; 2 School of Education, University of Rwanda College of Education (URCE), Kayonza, Rwanda

**Keywords:** Google Scholar, framework, mathematics, pedagogical content knowledge, technological pedagogical and content knowledge

## Abstract

Background: A study to reveal existing pedagogical content knowledge or technological pedagogical content knowledge frameworks and their effectiveness in teaching mathematics is crucial to inform the reader, teacher, and researcher. This review study intended to explore the trends of the pedagogical content knowledge (PCK) framework, how it has changed over time until the most recent version of technological and pedagogical content knowledge (TPACK) was developed, and their effectiveness in teaching mathematics.

Methods: We initially downloaded 273 articles from the first 30 Google Scholar pages and analyzed 229 journal articles. We got 24 frameworks from 64 journal articles since Shulman’s first model in 1986. About 52 out of 229 were mathematics studies. Among these studies, we found that 18 studies have extensively investigated the use of identified frameworks.

Results: The frameworks were presented and descriptively discussed in chronological order. The empirical studies that compared the role of pedagogical content knowledge and technological pedagogical content knowledge models among classrooms with teachers who possess and do not possess such skills were demonstrated.

Conclusions: The gap in empirical studies was identified, and further studies about the intervention of PCK and TPACK models were suggested to gain more insight into the mathematics classroom.

## Introduction

Around the 1980s, a new era in subject matter and teacher pedagogy rose. Shulman (1986) argues that the emphasis on teacher material knowledge and pedagogy is conserved as special. He believes that teacher training should conglomerate these two areas of specialization. He introduced the concept of pedagogical content knowledge (PCK), which comprises pedagogical knowledge (PK) and content knowledge (CK), to address this dichotomy, amongst other classifications. His original portrayal of the teacher's knowledge encompassed information about the curriculum and knowledge of the educational context.

After two decades, a major revolution has been proposed by various researchers in the field of technology. Mishara and Koehler (2006) established the concept of technological pedagogical and content knowledge (TPACK) in reaction to the lack of theory guiding technology integration into teaching. TPACK characterizes an extension of Shulman’s (1986) representation of what their colleagues looked for to teach explicit content (that is, PCK) by depicting the knowledge required to teach such content with technology ( Mishara & Koehler, 2006).

### Problem statement

The development of effective pedagogical strategies that integrate technology into the teaching of mathematics is a complex endeavor. In the landscape of educational research, a significant gap exists in understanding the evolution of PCK and TPACK frameworks, particularly within mathematics education. This gap becomes evident when considering the existing review articles found in the field. Our investigation revealed a review article from 2008 by Smith and Anagnostopoulos, which explored the development of PCK among English teachers within institutional networks. Conversely, a more recent review in 2020 by Njiku *et al.* focused on analyzing research instruments employed across 28 diverse studies to assess teacher TPACK. However, despite these contributions, a comprehensive and cumulative exploration of the evolution of PCK from its inception remains conspicuously absent in the literature.

In our analysis of 20 review articles, comprising 15 journal articles, two conference proceedings, two book chapters, and one book, we discovered a distinct lack of studies that systematically trace the progression of the PCK framework over time. Moreover, among the identified journal articles, none extensively examined teaching interventions facilitated by PCK and TPACK frameworks, nor did they provide a detailed investigation into the challenges teachers face when striving to acquire content knowledge. For instance, while notable efforts have been made to understand the methods and instruments of TPACK through studies by Abbitt (2011), Aydin and Boz (2012), and Young *et al.* (2012), a comprehensive examination of PCK’s development has been notably absent. Similarly, reviews conducted by Chai *et al.* (2013), Voogt *et al.* (2013), Depaepe *et al.* (2013), and Wu (2013) have examined aspects of TPACK, but the narrative of PCK’s evolution remains incomplete.

Furthermore, individual articles such as the critical review by HU (2014), the analysis of TPACK studies in Turkey by Yilmaz (2015), and the work by Rosenberg and Koehler (2015) have contributed unique perspectives to the field. However, the lack of a comprehensive investigation into the cumulative development of PCK hampers a holistic understanding of the subject. Similarly, while studies by Evens *et al.* (2016), Hubbard (2018), De Rossi and Trevisan (2018), and Grieser and Hendricks (2018) have provided valuable insights into specific aspects of PCK and TPACK, a coherent narrative spanning the entirety of PCK’s evolution is still missing. In light of these observations, it becomes apparent that the literature lacks a consolidated and chronological exploration of the development of PCK and TPACK frameworks in teaching mathematics. While previous review articles have contributed valuable insights, they have not collectively addressed the evolution of PCK over time. This research aims to bridge this gap by providing a comprehensive mapping review that traces the evolution of both PCK and TPACK frameworks within mathematics education. In doing so, we seek to enhance the overall understanding of how these frameworks have evolved, how they have been applied in teaching mathematics, and their effectiveness in acquiring content knowledge.

Therefore, our study aimed at reviewing:

1. Chronological trends in PCK models in teaching and learning mathematics (from beginning to date, how did researchers modify the original model that Shulman started with? Which models, what was modified, why were the modifications made, which teachers and/or students benefited from that change?)
2. Effectiveness of PCK and TPACK frameworks in teaching and learning mathematics (empirical studies that have used these frameworks to upgrade students learning. Any level, primary-secondary-university. Any year. Descriptive and inferential statistics each study used, etc.).

## Methodology

### Data selection

**Figure 1.**
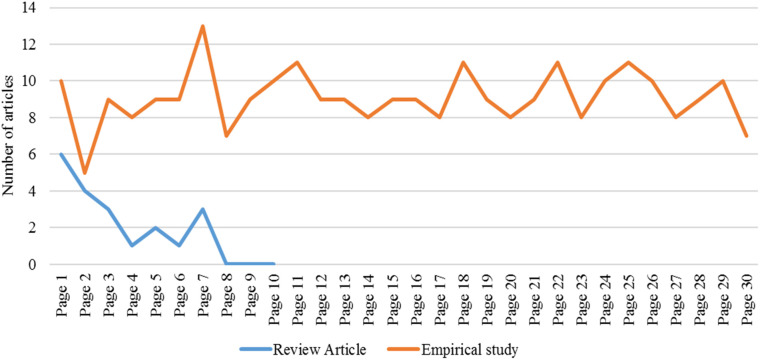
Number of studies downloaded from each page of Google Scholar.

We designed and employed a “one-term in one-source” method. This method involves using one term, a key, or search word in only one academic search engine. Thus, we have used “pedagogical content knowledge” as the search term in Google Scholar as an academic source. This search returned 20 review papers and 273 empirical studies at a glance (see Figure 1) on 19 August 2021. The choice of Google Scholar was influenced by its extensive coverage of scholarly articles and user-friendly interface facilitating efficient searches to access a wide array of academic publications.

Using the term “pedagogical content knowledge” in Google Scholar, we explored the results in the first 30 pages. We downloaded every article that contains the words “pedagogical content knowledge” in its title. We only included articles written in English. The first paper, *“Pedagogical content knowledge in social studies,”* was published in 1987, while the latest *“A virtual internship for developing technological pedagogical content knowledge”* was published in 2020. The first seven pages exhausted all articles written in review form, such as a review of literature, systematic review, meta-analysis, etc. This means that we checked pages eight to 10 and had no results and stopped checking after page 10. There were many more empirical studies (articles that investigate with primary data), so we limited the search to the 30th page (entry); as you can see, the last page still had seven articles (see Figure 1) and no mathematics-related paper was available. We did not limit ourselves to year of coverage, subject, or grades level. However, we intended to explore mathematical PCK explicitly. Thus, we retrieved all articles regardless of subject but then later excluded the non-mathematics articles. Literature review articles were visited only to frame our research problem, while empirical articles were focused on the analysis to answer our research objectives. The first author did initial screening and the co-authors checked the inclusion criteria.

### Extended sources and nature of selected articles

Although we used only one database, we found articles from other repositories. For instance, 24 articles were from the educational resources information center (ERIC), 17 from Academia, 13 from Research Gate, two from HAL ( *Hyper Articles en Ligne*), and two from Durham University. However, all these were accessed via Google Scholar. Thus, articles in Google Scholar will take you to the website they are hosted on or repository they are deposited on. About the nature of articles selected, 229 were identified as journal articles, 22 were conference proceedings, 8 were book chapters, 2 were books, and 5 were generics (articles with unclear identification). Therefore, our analysis only considered journal articles due to their rigorous peer-review process that ensures a higher degree of academic scrutiny and quality.

**Figure 2.**
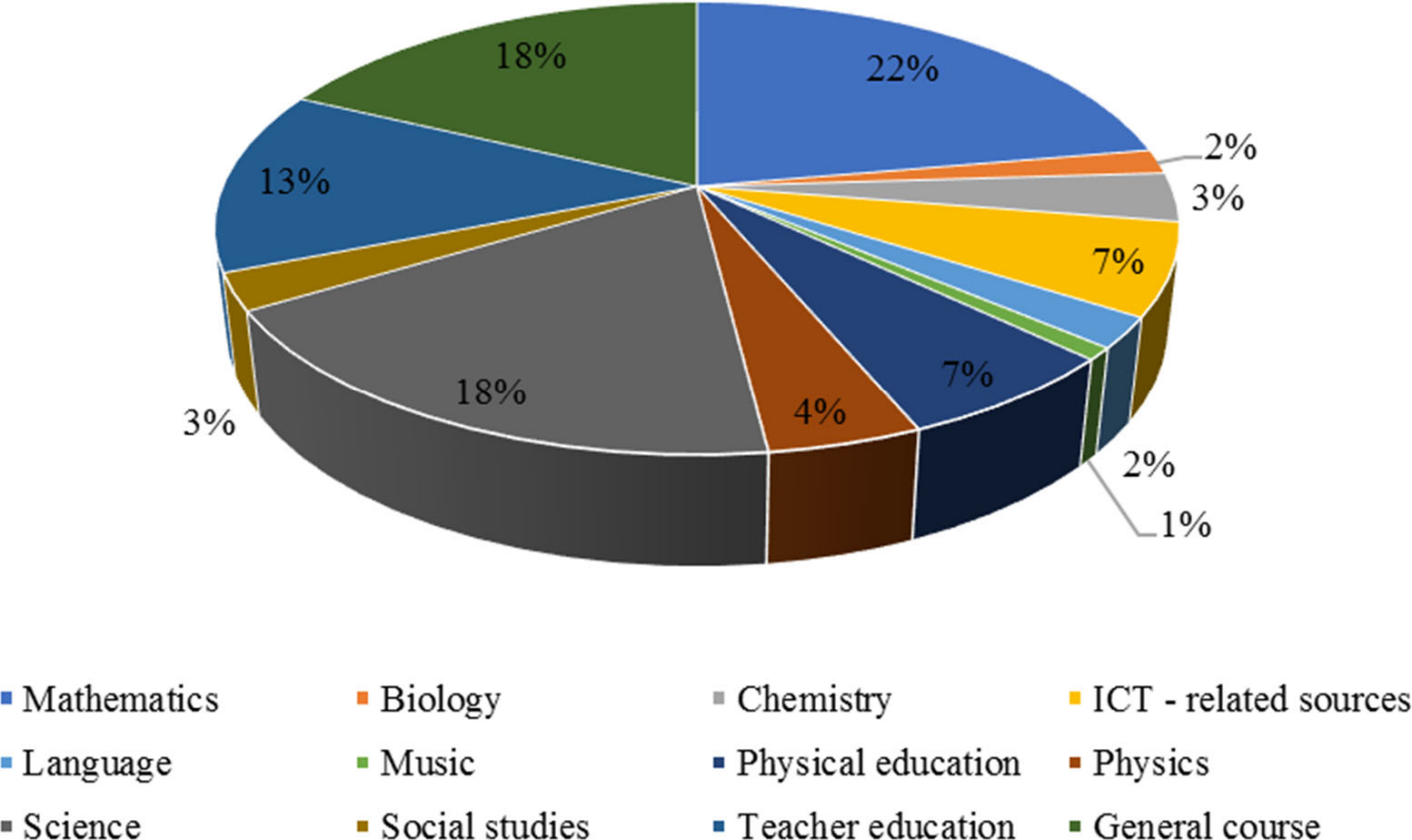
Subjects and number of articles.

12 subjects were identified across 229 articles. Articles discussing or investigating learning mathematics dominated the list (23% or 52 out of 229 articles). Science subjects and articles related to teacher education were 42 (18%) and 30 (13%) out of 229 articles, respectively, while unidentified articles or articles investigating PCK in a general sense accounted for 40 (17%). Figure 2 shows the distribution of articles among different subjects.

Among 229 retrieved journal articles, 110 (48%) were from 20 journals from various publishers. Table 1 shows that Teaching and Teacher Education, published by Elsevier, and the International Journal of Science Education, published by Taylor and Francis, were the two journals ranked first and second respectively among the top 20 contributors in this study (6% and 4%, respectively).

**Table 1.** Journals that mostly contributed to the present study.

	Journal	Publisher	Number of articles	% of articles
1	Teaching and Teacher Education	Elsevier B.V	13	6%
2	International Journal of Science Education	Tylor & Francis Ltd	10	4%
3	Computers & Education	Elsevier B.V	8	3%
4	Journal of Teacher Education	SAGE Publishing	8	3%
5	Journal of Teaching in Physical Education	Human Kinetics	8	3%
6	Australasian Journal of Educational Technology	Ascilite	6	3%
7	Journal of Research in Science Teaching	Wiley Periodicals	6	3%
8	Research in Science Education	Springer Publishing	6	3%
9	Contemporary Issues in Technology and Teacher Education	AACE	5	2%
10	International Journal of Science Education	Tylor & Francis Ltd	5	2%
11	Journal of Educational Computing Research	Baywood Publishing	5	2%
12	Journal of Science Teacher Education	Tylor & Francis Ltd	5	2%
13	Journal of Research in Science Teaching	Wiley Periodicals	4	2%
14	Educational Sciences: Theory & Practice	-	3	1%
15	Journal for Research in Mathematics Education	NCTM	3	1%
16	Journal of Digital Learning in Teacher Education	Tylor & Francis Ltd	3	1%
17	Journal of Research on Technology in Education	Tylor & Francis Ltd	3	1%
18	Research in Science & Technological Education	Tylor & Francis Ltd	3	1%
19	Research Quarterly for Exercise and Sport	Tylor & Francis Ltd	3	1%
20	Teachers and Teaching: theory and practice	Tylor & Francis Ltd	3	1%
			110	48%

AACE: Association for the Advancement of Computing in Education, NCTM: National Council of Teachers of Mathematics.

**Figure 3.**
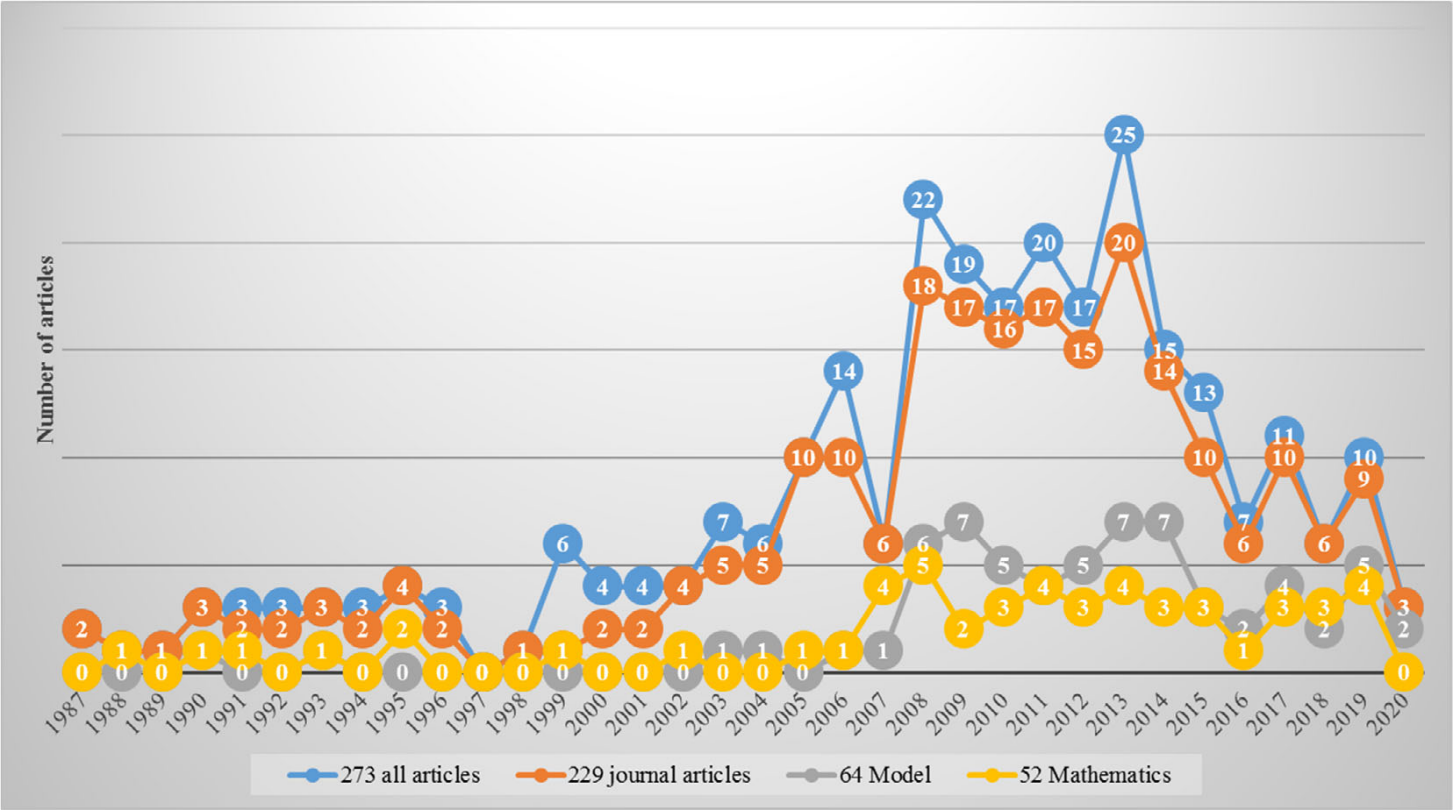
Displaying the hierarchy of downloaded articles.

Figure 3 displays the hierarchy of downloaded articles. The blue color chart shows the number of all empirical studies downloaded across the years of publication. The first study that investigated PCK, after its launch from Shulman in 1986, was published in 1987, while the latest came out in 2020. After filtering out proceedings, books, book chapters, theses, and generics, 229 journal articles are shown in red. After analyzing these 229 articles, we found 64 articles that clearly investigated or used PCK or TPACK frameworks or models (see grey color). Thus, others investigated these models. They did not formulate new models but used existing ones. Finally, among 229 articles, 52 articles were found to extensively investigate mathematics lessons, as shown in yellow.

### Data analysis

**Figure 4.**
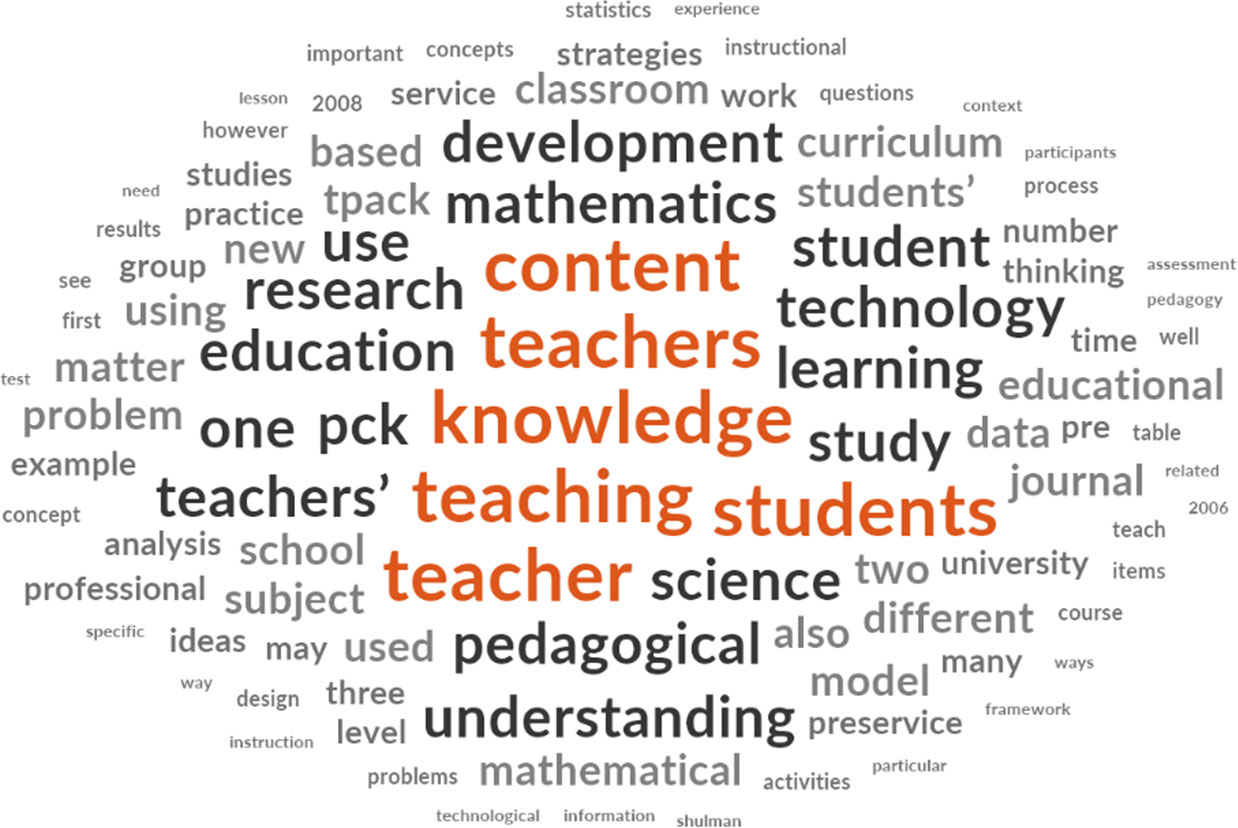
Word frequency from all downloaded articles.

To reveal trends of the PCK framework, we identified the year of publication, author of the study, model of PCK, and construct (whether PCK or TPACK) for each study. To reveal the effectiveness of PCK and TPACK interventions, we identified the construct, theoretical framework used to frame the study, research design, teaching intervention, topic, and analysis method for each study. PCK models were cited, presented in figures, and described. Teaching interventions were presented in sections, and their statistics were presented in tables and descriptively discussed. Teachers’ knowledge was presented descriptively or in tables by discussing the type of study (survey, cases studies, interventions, etc.) that produced the results, the type of data such as quantitative or qualitative, the type of mode such as whether teachers’ knowledge was measured from him/herself or from his/her students, etc. Theoretical strands were also described accordingly. We used both NVivo 1.0 software and MS Excel 2016 to analyze data. Figure 4 is a typical example of the word analysis from NVivo using articles that were downloaded.

## Results and discussion

### PCK framework

65 (including 22 related to mathematics) out of 229 showed PCK trend models. About 24 articles (including 11 related to mathematics) showed original frameworks, while 41 referred to these 24 articles. Thus, Table 2 displays a review of 24 frameworks presented in the related 24 articles. Among these 24 frameworks, 17 are versions of PCK, and seven are upgrades of TPACK.

**Table 2.** Trends of pedagogical content knowledge (PCK) framework.

	Year	Authors	Model	Construct
1	1990	( Marks, 1990)	A structure for PCK in fifth-grade equivalence of fractions	PCK
2	1993	( Geddis, 1993)	Transforming subject-matter knowledge	PCK
3	1995	( Simon, 1995)	Mathematics Teaching Cycle	PCK
4	2003	( Jones & Moreland, 2003)	7 component of framework	PCK
5	2004	( An *et al.*, 2004)	The network of PCK	PCK
6	2005	( Niess, 2005)	Four different aspects that comprise teachers’ TPCK	TPACK
7	2006	( Major & Palmer, 2006)	The PCK change process	PCK
8	2006	( Mishara & Koehler, 2006)	Pedagogical Technological Content Knowledge	TPACK
9	2008	( Hill *et al.*, 2008)	Domain map for mathematical knowledge for teaching	PCK
10	2008	( De Miranda, 2008)	Infusion of Engineering Knowledge, Pedagogy and Context in Technology Education Instruction	TPACK
11	2009	( Kaya, 2009)	Components of the PSTs’ PCK investigated	PCK
12	2009	( Koehler & Mishra, 2009)	The TPACK framework and its knowledge components	TPACK
13	2010	( Etkina, 2010)	The Structure of Physics Teacher Knowledge	PCK
14	2011	( Saeli *et al.*, 2011)	Diagram based on Grossman’s reformulation of PCK	PCK
15	2011	( Tee & Lee, 2011)	Key design considerations for creating activities and conditions to facilitate socialisation, externalisation, combination and internalisation	TPACK
16	2011	( Voss *et al.*, 2011)	Conceptualization of general pedagogical/psychological knowledge (PPK)	PCK
17	2012	( McCray & Chen, 2012)	Pedagogical content knowledge	PCK
18	2012	( Saeli *et al.*, 2012)	Scheme representing the relation between Saeli *et al.*’s (2012) study and their previous ones	PCK
19	2013	( Zepke, 2013)	Revisiting PCK	PCK
	2013	( Lannin *et al.*, 2013)	A model of PCK for teaching mathematics	PCK
20	2015	( Olfos *et al.*, 2014)	Structures of the studies of the teacher’s PCK and CK	PCK
21	2019	( Gess-Newsome *et al.*, 2019)	Project PRIME theoretical path of influence/Model of teacher professional knowledge and skill including PCK	PCK
22	2019	( Chai *et al.*, 2019)	The revised scaffolded TPACK lesson design model (R-STLDM)	TPACK
23	2020	( Gasteiger *et al.*, 2020)	Explicit and implicit knowledge of early childhood teachers	PCK
24	2020	( Andyani *et al.*, 2020)	The TPACK component and extract the structural model of the mutual relationship between its constructs	TPACK

Table 2 outlines the models and constructs for each study. We are now going to discuss each of the 24 concepts in summary.

1. Nature and Components of PCK Frameworks ( Jones & Moreland, 2003; McCray & Chen, 2012; Lannin *et al.*, 2013)
  These foundational studies unveil the multifaceted nature of PCK by highlighting its core constructs. Jones and Moreland (2003) identified seven pivotal components, including understanding the subject nature, curriculum knowledge, and awareness of students’ prerequisites. McCray and Chen (2012) extended this understanding, segregating PCK into subject matter comprehension, knowledge of students’ development, and effective teaching methodologies. Lannin *et al.* (2013) expanded the framework to encompass knowledge of mathematics curriculum, instructing techniques, assessment methods, and students’ comprehension of mathematics. Collectively, these works underscore the comprehensive nature of PCK, illuminating its role in effective mathematics instruction.
2. PCK’s Role in Addressing Misconceptions and Enhancing Learning ( Geddis, 1993; Olfos *et al.*, 2014; Etkina, 2010)
Geddis (1993) introduced the notion of PCK as a tool to remedy students’ misconceptions, emphasizing its role in utilizing alternative representations to foster understanding. This theme resonates in Olfos *et al.*’s (2014) examination of teacher knowledge, where PCK is identified as a vehicle to adapt content, remediate learning difficulties, and develop nuanced assessment strategies. Etkina’s (2010) model further reinforces this by spotlighting PCK's contribution to cultivating effective teaching strategies, assessment techniques, and strategies to comprehend students' needs. Collectively, these studies paint a vivid picture of PCK as a powerful mechanism to rectify misconceptions and elevate learning outcomes in mathematics.
3. The Dynamic Interaction of PCK with Other Knowledge Domains ( Koehler & Mishra, 2009; Gess-Newsome *et al.*, 2019; Andyani *et al.*, 2020)
  The interplay between PCK and other knowledge domains, as evident in Koehler and Mishra’s (2009) Technological Pedagogical Content Knowledge (TPACK) framework, introduces a more holistic perspective. This interconnectedness is further highlighted by Gess-Newsome *et al.* (2019), who depict PCK as one of the pillars of teachers’ professional knowledge alongside content, pedagogical, assessment, and student knowledge. Additionally, Andyani *et al.* (2020) assert that PCK's convergence with content and technological knowledge culminates in the comprehensive Technological Pedagogical and Content Knowledge (TPACK). These works collectively underscore the intricate interplay of PCK with other dimensions of teachers’ expertise.
4. Examining PCK’s Influence on Student Learning ( Marks, 1990; Simon, 1995; Chai *et al.*, 2019)
Marks (1990) explored PCK’s influence on student learning by emphasizing its interconnectedness with instructional processes, student focus, and media. Simon (1995) further contributed to this understanding by highlighting the pivotal role of teachers’ capacity to hypothesize students’ knowledge and tailor learning activities accordingly. Chai *et al.*’s (2019) model advances this by mapping PCK within the Revised Scaffolded TPACK Lesson Design Model, linking it intricately with instructional and learning activities. Collectively, these studies illuminate how PCK shapes instructional practices and learning trajectories to maximize student learning outcomes.

### Synthesis and implications

Cumulatively, the studies reviewed here weave a tapestry of insights that converge on the significance of Pedagogical Content Knowledge (PCK) in mathematics education. PCK serves as a multifaceted framework that encompasses not only subject matter comprehension and teaching strategies but also the vital role of remedying misconceptions, addressing diverse student needs, and integrating technology into instruction. As PCK converges with content and technological knowledge, it becomes a key element within broader constructs like TPACK, highlighting the intricate network of expertise required for effective teaching.

Furthermore, these studies collectively emphasize the dynamic nature of PCK’s interaction with other knowledge domains and its transformative role in shaping student learning trajectories. The synthesis of these insights underscores the integral role of PCK in scaffolding effective mathematics instruction, aligning pedagogical approaches with curriculum objectives, student characteristics, and diverse learning contexts.

### The effectiveness of using PCK and TPACK interventions in Mathematics education

52 out 229 were identified as articles related to mathematics. Among 52 mathematics articles, 39 were related to TPACK, while 13 were investigating PCK. 18 of the 52 articles fit our analysis framework and spanned from 1988 to 2020. They showed the implications of PCK and TPACK frameworks. Except for these 18 articles, 34 out of 52 articles reviewed showed survey results that only provided the outcome of PCK or TPACK. These 18 articles were only articles that investigated the outcome of comparative studies such as pre-and post-test designs, variables such as teachers’ backgrounds, teachers’ gender, teachers’ geographical locations, etc. Most of the studies left out were surveys without comparison, and others were qualitative. Table 3 displays a review of 18 articles related to mathematics that showed the comparative outcome of PCK and TPACK.

**Table 3.** Effectiveness of PCK and TPACK interventions.

	Study	Construct	Theoretical framework	Research design	Teaching intervention	Topic	Analysis
1	Teachers’ PCK of students’ problem-solving in elementary arithmetic ( Carpenter *et al.*, 1988)	PCK	-	Survey, correlation	The study compared teachers’ estimates and students’ performance	Functions	Quantitatively descriptive
2	The Lesson Preparation Method: a way of investigating pre-service teachers’ PCK ( Van Der Valk & Broekman, 1999)	PCK	-	Survey	Lesson preparation task and the subsequent interview	Area	Qualitatively descriptive
3	Preparing teachers to teach science and mathematics with technology: Developing a technology PCK ( Niess, 2005)	TPCK	Constructivism	Pre and post-assessment	Microteaching	-	Qualitatively descriptive
4	Development of mathematics PCK in student teachers ( Lim-Teo *et al.*, 2007)	PCK	-	Pre and post-testing	Methodology course	-	Inferential statistics
5	Preparing to teach mathematics with technology: an integrated approach to developing TPACK ( Lee & Hollebrands, 2008)	TPACK	-	Quasi-experiment	Technology and an Integrated Approach	-	Inferential statistics
6	Secondary mathematics teachers’ PCK and content knowledge: validation of the COACTIV constructs ( Krauss, Baumert, *et al.*, 2008)	PCK	Theory of adult intellectual development	Teacher-students comparative study	Cognitive Activation in the Classroom (COACTIV) approach with related conceptualizations	-	Inferential statistics
7	Teaching area and perimeter: mathematics PCK in-action ( Kow & Yeo, 2008)	PCK	-	Beginning and senior teacher comparative study, classroom observation, and field notes	Lesson delivery	Area and perimeter	Qualitatively descriptive
8	PCK and content knowledge of secondary mathematics teachers ( Krauss, Brunner, *et al.*, 2008)	PCK	Generalizability theory	Correlation study	In-depth mathematical training	-	Inferential statistics
9	Mathematics teachers’ topic-specific PCK in the context of teaching a ^0^, 0! and a ÷ 0	PCK	-	Comparative study between experienced and novice teachers	-	a ^0^, 0! And ÷0	Deductive content analysis
10	From socialization to internalization: cultivating TPACK through problem-based learning ( Tee & Lee, 2011)	TPACK	-	Prior and after the course for Mathematics and Education groups	Proto-theories in the form of a problem-based learning approach guided by the socialization, externalization, combination, and internalization framework	-	Inferential statistics
11	Teacher education effectiveness: quality and equity of future primary teachers’ mathematics and mathematics PCK ( Blömeke *et al.*, 2011)	PCK	Education and social inequality	Language and gender differences in mathematics CK and mathematics PCK	-	-	Inferential statistics
12	Teachers’ content knowledge and PCK: the role of structural differences in teacher education ( Kleickmann *et al.*, 2013)	PCK	-	Cross-sectional data from four samples, pre-and in-service teachers	Cognitive Activation in the Classroom (COACTIV)	-	Inferential statistics
13	Teachers’ PCK and its relation with students’ understanding ( Olfos *et al.*, 2014)	PCK	Vergnaud’s theory of conceptual fields	An ex-post-facto non-experimental design implemented with ten variables	-	-	Inferential statistics
14	Integrating PCK and pedagogical/psychological knowledge in mathematics ( Harr *et al.*, 2014)	PCK	Bandura sub-theory on abstract modelling	Pre- and post-test design	Computer-based working memory task	-	Inferential statistics
15	Teachers’ content and PCK on rational numbers: A comparison of prospective elementary and lower secondary school teachers ( Depaepe *et al.*, 2015)	PCK	The theory of conceptual change	Pre- and in-service teachers assessed among elementary and lower secondary school teachers	Three years of professional training	Rational numbers	Inferential statistics
16	Content knowledge and PCK in Taiwanese and German mathematics teachers ( Kleickmann *et al.*, 2015)	PCK	-	Cross-sectional study and data from a sample of experienced in-service and experienced teachers	-	Algebra, Geometry	Inferential statistics
17	TPACK of mathematics teachers and the effect of demographic variables ( Ozudogru & Ozudogru, 2019)	TPACK	-	Survey design, Multivariate and Univariate analyses for gender, level of school, and teaching experience	-		Quantitatively descriptive
18	Decolonising TPACK of first-year mathematics students ( Khoza & Biyela, 2020)	TPACK	A unified theory of acceptance and use of technology	Survey and post-observation	GeoGebra resources	Algebra, Trigonometry	Qualitatively describe

Table 3 presents PCK and TPACK framework interventions. We are now going to discuss them in more detail and reveal their effectiveness in teaching mathematics. Within the realm of mathematics education, the exploration of PCK and TPACK interventions has yielded a plethora of studies that collectively shed light on their effectiveness. As we delve into this landscape, we will traverse a spectrum of research endeavors that have examined these frameworks’ implications for educators and learners.

1. Enhancing Problem-Solving Instruction ( Carpenter *et al.*, 1988)
  Carpenter and colleagues ventured into the pedagogical content of grade-one teachers, scrutinizing their grasp of children's explanations of word problems in mathematics. The study underscored the significance of teachers' ability to comprehend critical differences in students' problem-solving approaches. This insight laid the groundwork for a deeper exploration of how effective teaching strategies can be aligned with diverse problem-solving challenges.
2. Fostering Pre-Service Teachers' PCK ( Van Der Valk & Broekman, 1999)
  The investigation led by Van Der Valk and Broekman proposed a methodological approach for nurturing pre-service teachers' PCK. By urging trainee teachers to formulate lessons and harness their subject matter knowledge, the study pointed to the efficacy of engaging educators in designing and planning instructional activities. This approach echoed the idea that cultivating PCK can be a catalyst for effective teaching preparation.
3. Integrating Technology within TPACK ( Niess, 2005)
  Niess delved into the intricate fusion of PCK and technology, presenting the Technological Pedagogical Content Knowledge (TPACK) concept. Through a multidimensional training program for science and math teachers, the study showcased the evolving nature of PCK when infused with technology. This intervention highlighted the need to integrate subject matter knowledge, pedagogical insights, and technological tools seamlessly.
4. Unveiling PCK Development ( Lim-Teo *et al.*, 2007)
  Lim-Teo and colleagues probed the development of Mathematics PCK (MPCK) in student teachers, emphasizing the transformative impact of pedagogical courses. Their work illuminated the evolution of teachers' knowledge in mathematics teaching, showcasing the positive impact of targeted pedagogy-focused training on bolstering PCK.
5. PBL-Based Approach to Cultivate TPACK ( Tee & Lee, 2011)
  Tee and Lee advocated for a Problem-Based Learning (PBL) approach as a conduit to foster Technological Pedagogical Content Knowledge (TPACK). Their study depicted how a classroom environment fostering socialization, externalization, pairing, and internalization facilitated teachers in harnessing TPACK more effectively. This finding underscored the significance of well-structured educational environments in nurturing TPACK.
6. Global Perspectives on Teacher Education Effectiveness ( Blömeke *et al.*, 2011)
  Blömeke and team embarked on a cross-cultural journey to assess the effectiveness of primary teacher training programs across 15 nations. The study unraveled a landscape of disparities, demonstrating the nuanced influences of cultural contexts on the outcomes of teacher education. This exploration broadened the understanding of PCK efficacy in diverse global settings.

### Synthesis and implications

These studies weave a tapestry of insights into the efficacy and impact of PCK and TPACK interventions in mathematics education. Through these lenses, educators have sought to enhance problem-solving strategies, leverage technology to bolster teaching, and design comprehensive teacher education programs. While PCK and TPACK serve as pivotal frameworks, the studies collectively underline the need for a holistic approach to teaching mathematics. The recurring themes of knowledge integration, contextual relevance, and dynamic instructional methodologies transcend individual studies. However, gaps also emerge, particularly in integrating certain framework components. While PCK and TPACK offer valuable lenses, the studies suggest that they might not be the sole factors accounting for effective learning outcomes in mathematics education. The intricate interplay of content, pedagogy, and technology must align with diverse learner characteristics and changing educational landscapes.

## Conclusion

**Figure 5.**
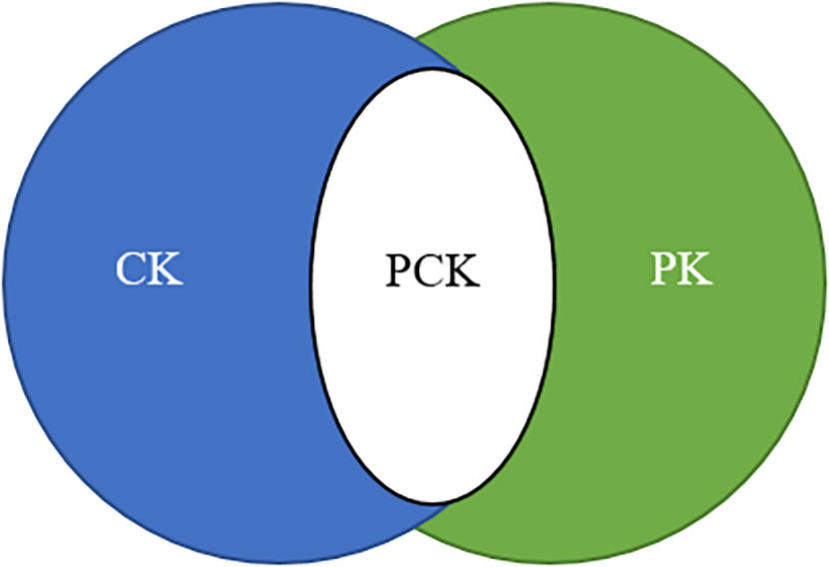
Original PCK framework, from Shulman (1986 and 1987).

In this study, we employed a focused method that proved effective in delving into the literature and extracting the relevant information necessary to address our research questions. The genesis of the concept of pedagogy’s centrality in delivering effective lessons dates back to the early 1980s when Shulman (1986) laid the foundation. He advocated for an expanded view of a teacher's requisite knowledge, emphasizing the significance of content knowledge and pedagogical knowledge, including curriculum, students, and instructional insights ( Shulman, 1987). Shulman’s framework, as depicted in Figure 5, has since spurred a research continuum. The framework underwent adaptations to accommodate different contexts, ranging from diverse subjects to integrating information and communication technology (ICT). This dynamic evolution culminated in the introduction of the technological, pedagogical, content knowledge (TPCK) constructs, later streamlined as the technological, pedagogical, and content knowledge (TPACK) model, reflecting the increasing role of technology in 21st-century education ( Mishra & Koehler, 2008).

Our study, driven by the aspiration to address a gap in the literature, delved into the hierarchical modifications of these frameworks across various teaching contexts. Specifically, we sought to discern their impact on refining mathematics education by analyzing empirical studies that employed PCK and TPACK frameworks as interventions to enhance learning outcomes. Our analysis revealed a pronounced emphasis on PCK, with 13 out of 18 reviewed papers centering around this construct. However, while these studies primarily surveyed teachers’ performance in distinct components of these frameworks, few delved into fostering PCK and TPACK skills to enhance students’ conceptual understanding, attitudes, and performance. Consequently, a pressing need exists to further explore these skills within the classroom context and their resultant impact on student learning. One notable area for future investigation is the comparison between teachers equipped with strong PCK skills and those without, discerning the ripple effects on their students’ outcomes.

A pivotal avenue of research should encompass teachers’ challenges in acquiring either content knowledge (CK) or pedagogical knowledge (PK), dissecting the nuances through empirical studies encompassing surveys, case studies, and interventions. The comprehensive illumination of teacher knowledge, regardless of its form, can guide pedagogical practices and elevate educational outcomes. In summation, our study’s trajectory has elucidated the dynamic evolution of PCK and TPACK frameworks, prompting a proliferation of research that has, in turn, informed the landscape of effective teaching and learning strategies. As we move ahead, an agenda ripe with opportunities for uncovering the intricate relationship between these constructs, teacher capabilities, and student achievements emerges. The journey into the depths of effective education continues, with the tools of PCK and TPACK guiding us toward a brighter horizon of pedagogical excellence and improved learning outcomes.

## Data availability

All data underlying the results are available as part of the article and no additional source data are required.

## Author contribution

All authors have equally participated in designing and communicating the results.
