# Supplementary material for: From pedagogical content knowledge toward technological pedagogical content knowledge frameworks and their effectiveness in teaching mathematics: A mapping review
Source: F1000Res. 2023 Aug 17;11:1029. Originally published 2022 Sep 12. [Version 2] doi: 10.12688/f1000research.125073.2 (PMC10521045; doi:10.12688/f1000research.125073.2)
Supplement: Peer review history

# Reviewer report: Reviewer response for version 2

**DOI:** 10.5256/f1000research.154567.r197664

**Published:** 2023-09-15

**Authors:** Joseph Njiku

**Affiliations:** 1 Dar es Salaam University College of Education, University of Dar es Salaam, Dar es Salaam, Tanzania

It is now OK.

Is the work clearly and accurately presented and does it cite the current literature?

Partly

If applicable, is the statistical analysis and its interpretation appropriate?

Not applicable

Are all the source data underlying the results available to ensure full reproducibility?

Yes

Is the study design appropriate and is the work technically sound?

Yes

Are the conclusions drawn adequately supported by the results?

Partly

Are sufficient details of methods and analysis provided to allow replication by others?

Partly

Reviewer Expertise:

Mathematics education, teacher education, and technology integration in education.

I confirm that I have read this submission and believe that I have an appropriate level of expertise to confirm that it is of an acceptable scientific standard.

# Reviewer report: Reviewer response for version 2

**DOI:** 10.5256/f1000research.154567.r197663

**Published:** 2023-08-29

**Authors:** Jones Apawu

**Affiliations:** 1 Department of Mathematics Education, University of Education Winneba, Winneba, Central, Ghana

I have read the revised version of the article and can say that the authors have taken into consideration the concerns that were raised.

Is the work clearly and accurately presented and does it cite the current literature?

Yes

If applicable, is the statistical analysis and its interpretation appropriate?

Yes

Are all the source data underlying the results available to ensure full reproducibility?

Yes

Is the study design appropriate and is the work technically sound?

Yes

Are the conclusions drawn adequately supported by the results?

Yes

Are sufficient details of methods and analysis provided to allow replication by others?

Yes

Reviewer Expertise:

Technology integration into the teaching of mathematics, Algebraic thinking of learners, Differentiated instructions

I confirm that I have read this submission and believe that I have an appropriate level of expertise to confirm that it is of an acceptable scientific standard.

# Reviewer report: Reviewer response for version 1

**DOI:** 10.5256/f1000research.137338.r186613

**Published:** 2023-08-03

**Authors:** Jones Apawu

**Affiliations:** 1 Department of Mathematics Education, University of Education Winneba, Winneba, Central, Ghana

After reading the article, which is a mapping review, there seems to be no pedagogical framework but rather PCK and TPACK frameworks. I suggest “From pedagogical content knowledge toward technological pedagogical content knowledge frameworks and their effectiveness in teaching mathematics: A mapping review. The article tried to give out the trend in both PCK and TPACK frameworks usage. The paper would be good for researchers who want to employ PCK and TPACK as their theoretical framework. However, there are some concerns under the following headings that I suggest the authors should look at.

**Abstract**

 “Pedagogical or technological pedagogical content knowledge frameworks” seems as if pedagogical and TPACK are the same. Also, is crucial to inform what? It must be stated explicitly. So, this sentence “A study to reveal existing pedagogical or technological pedagogical content knowledge frameworks is crucial to inform and their effectiveness in teaching mathematics” needs recasting. “… and its effectiveness in teaching mathematics” is not specific. Is it PCK’s or TPACK’s effectiveness…? The last sentence under Background should be relooked at since it is not in relation with mathematics. The first sentence under Results, “The empirical studies that compared the role of pedagogical and technological pedagogical content knowledge model… seems like we have pedagogical model as well.

**Problem statement**

 Rossi and Trevisan (2018) investigated how TPCK is defined… seems like the authors are claiming Rossi and Trevisan (2018) tried to define TPCK. In 2005, it was TPCK and changed to TPACK in 2007 and context was added in 2008. The authors may wish to revisit the literature. The last sentence of 2 (see page 3), “Descriptive and inferential statistics each study got, etc.). Is it each study used or got? Look at that again.

**Methodology**

 The method “ *one-term in one-source”* (see page 3) is not well known to the research community. You may wish to look at it again. The sentence, “There were many more empirical studies (articles that investigate with primary data), so we limited the search to the 30th page (entry); as you can see, the last page still had seven articles.” (see around line 5 under Figure, page 4) Should read “There were many more empirical studies (articles that investigate with primary data), so we limited the search to the 30th page (entry); as you can see, the last page still had seven articles (see Figure 1).

**Data analysis**

 In Figure 4, the green colour is not showing well. It looks like grey colour. The authors referred to it as green colour (see sentence 3 under Figure 4, page 6)

**Results and discussion**

- On page 7, the first sentence under Table 2 should rather read the summary of the articles and not … in more detail.
- The authors can consider looking at articles that relate to mathematics teaching as the title of the paper depicts.
- On page 8, the first sentence under “Seven components of the framework”, from Table 2, it should be authors and not author.
- On page 8, in the first sentence under “Components of the PSTs’ PCK investigated”, from Table 2, it should be author and not authors.
- The effectiveness of using the frameworks did not show explicitly under “PCK interventions” as the title of the paper depicts.
- The discussions were not done in relation to literature by connecting articles that use the frameworks to enhance effective teaching of mathematics and those that did not.

**References**

 The following cited works are not on the references list:

- Rossi and Trevisan (2018)
- Miranda (2008)

The following references were not cited:

- De Miranda MA: Pedagogical Content Knowledge and Technology Teacher Education: Issues for thought. Journal of the Japanese Society of Technology Education. 2008; 50(1): 17–26.
- De Rossi M, Trevisan O: Technological Pedagogical Content Knowledge in the literature: how TPCK is defined and implemented in initial teacher education Technological Pedagogical Context Knowledge (TPCK) in letteratura: come viene definito e implementato il modello TPCK nei conte. Ital. J. Educ. Technol. 2018; 26(1): 7–23.

Is the work clearly and accurately presented and does it cite the current literature?

Yes

If applicable, is the statistical analysis and its interpretation appropriate?

Yes

Are all the source data underlying the results available to ensure full reproducibility?

Yes

Is the study design appropriate and is the work technically sound?

Yes

Are the conclusions drawn adequately supported by the results?

Yes

Are sufficient details of methods and analysis provided to allow replication by others?

Yes

Reviewer Expertise:

Technology integration into the teaching of mathematics, Algebraic thinking of learners, Differentiated instructions

I confirm that I have read this submission and believe that I have an appropriate level of expertise to confirm that it is of an acceptable scientific standard, however I have significant reservations, as outlined above.

## References

[1] : Pedagogical Content Knowledge and Technology Teacher Education: Issues for thought. *Journal of the Japanese Society of Technology Education* .2008;1(50) :17-26

[2] : Technological Pedagogical Content Knowledge in the literature: how TPCK is defined and implemented in initial teacher education. *Italian Journal of Educational Technology* .2018;1(26) :7-23

# Response

**Published:** 2023-08-11

**Authors:** Hashituky Telesphore Habiyaremye

**Affiliations:** University of Rwanda, Rwanda

**Reviewer comments:**

 After reading the article, which is a mapping review, there seems to be no pedagogical framework but rather PCK and TPACK frameworks. I suggest “From pedagogical content knowledge toward technological pedagogical content knowledge frameworks and their effectiveness in teaching mathematics: A mapping review. The article tried to give out the trend in both PCK and TPACK frameworks usage. The paper would be good for researchers who want to employ PCK and TPACK as their theoretical framework. However, there are some concerns under the following headings that I suggest the authors should look at.

**Author response: Thank you so much for your guidelines. We have adapted to the suggested title by including “…content knowledge…”**

**Abstract** “Pedagogical or technological pedagogical content knowledge frameworks” seems as if pedagogical and TPACK are the same. Also, is crucial to inform what? It must be stated explicitly. So, this sentence “A study to reveal existing pedagogical or technological pedagogical content knowledge frameworks is crucial to inform and their effectiveness in teaching mathematics” needs recasting. “… and its effectiveness in teaching mathematics” is not specific. Is it PCK’s or TPACK’s effectiveness…? The last sentence under Background should be relooked at since it is not in relation with mathematics. The first sentence under Results, “The empirical studies that compared the role of pedagogical and technological pedagogical content knowledge model… seems like we have pedagogical model as well.

**Author response: The first sentence of the background was changed as follows: “pedagogical” was replaced by “pedagogical content knowledge”, and “is crucial to inform and their effectiveness in teaching mathematics” was replaced by “and their effectiveness in teaching mathematics is crucial to inform reader, teacher and researcher.” The last sentence under background was changed as follows” “its” was replaced by “their.”**

**We added “content knowledge” after “pedagogical” in the sentence under results.**

**Problem statement **Rossi and Trevisan (2018) investigated how TPCK is defined… seems like the authors are claiming Rossi and Trevisan (2018) tried to define TPCK. In 2005, it was TPCK and changed to TPACK in 2007 and context was added in 2008. The authors may wish to revisit the literature. The last sentence of 2 (see page 3), “Descriptive and inferential statistics each study got, etc.). Is it each study used or got? Look at that again.

**Author response: The whole section was revisited to ensure clarity. “got” was changed to “used.”**

**Methodology **The method “one-term in one-source” (see page 3) is not well known to the research community. You may wish to look at it again. The sentence, “There were many more empirical studies (articles that investigate with primary data), so we limited the search to the 30th page (entry); as you can see, the last page still had seven articles.” (see around line 5 under Figure, page 4) Should read “There were many more empirical studies (articles that investigate with primary data), so we limited the search to the 30th page (entry); as you can see, the last page still had seven articles (see Figure 1).

**Author response: ​​​​​​​We invented the “one-term in one source” method.**

**We changed the sentence as you suggested: "There were many more empirical studies (articles that investigate with primary data), so we limited the search to the 30th page (entry); as you can see, the last page still had seven articles (see Figure 1)."**

**Data analysis** In Figure 4, the green colour is not showing well. It looks like grey colour. The authors referred to it as green colour (see sentence 3 under Figure 4, page 6)

**Author response: ​​​​​​​Thank you! The grey replaced green, and the yellow replaced purple.**

**Results and discussion**

 ○ On page 7, the first sentence under Table 2 should rather read the summary of the articles and not … in more detail.

 ○ The authors can consider looking at articles that relate to mathematics teaching as the title of the paper depicts.

 ○ On page 8, the first sentence under “Seven components of the framework”, from Table 2, it should be authors and not author.

 ○ On page 8, in the first sentence under “Components of the PSTs’ PCK investigated”, from Table 2, it should be author and not authors.

 ○ The effectiveness of using the frameworks did not show explicitly under “PCK interventions” as the title of the paper depicts.

 ○ The discussions were not done in relation to literature by connecting articles that use the frameworks to enhance effective teaching of mathematics and those that did not.

**Author response:**

**○​​​​​​​“summary” replaced “in detail.”**

**○​​​​​​​Papers discussing mathematics teaching were identified in Tables 2 and 3.**

**○​​​​​​​We added “s” on “author.”**

**○​​​​​​​“The effectiveness of using…” was added to the “PCK interventions.”**

**○​​​​​​​We removed “s” on “authors.”**

**○​​​​​​​The discussion connected articles instead of discussing each article separately.**

**References **The following cited works are not on the references list:

 ○ Rossi and Trevisan (2018)

 ○ Miranda (2008) The following references were not cited: De Miranda MA: Pedagogical Content Knowledge and Technology Teacher Education: Issues for thought. Journal of the Japanese Society of Technology Education. 2008; 50(1): 17–26.

 ○ De Rossi M, Trevisan O: Technological Pedagogical Content Knowledge in the literature: how TPCK is defined and implemented in initial teacher education Technological Pedagogical Context Knowledge (TPCK) in letteratura: come viene definito e implementato il modello TPCK nei conte. Ital. J. Educ. Technol. 2018; 26(1): 7–23.

**Author response: There was an error in writing these names, Miranda instead of De Miranda and Rossi instead of De Rossi**

# Reviewer report: Reviewer response for version 1

**DOI:** 10.5256/f1000research.137338.r153309

**Published:** 2022-11-07

**Authors:** Joseph Njiku

**Affiliations:** 1 Dar es Salaam University College of Education, University of Dar es Salaam, Dar es Salaam, Tanzania

**Summary:** the study is a literature review. It is designed to discuss the trend in PCK and TPACK studies and how they influence effective learning, especially in mathematics. The study reviews different literature in the area to respond to two research questions. Data sources are obtained from the Google Scholar database and screened for relevance. Data analysis is done to uncover the trends in PCK and TPACK and their effectiveness in explaining learning. As such findings are presented first by listing and explaining studies that explain the PCK or TPACK framework and those that explain the use of the framework in explaining learning especially in mathematics. The topic is current and may inform future studies on the evolution and debate in PCK and TPACK and how they have been used to influence learning. Specific comments about possible areas for improvement of the paper are provided as follows:

**Keywords:** although the authors used the Google Scholar database as the source of their articles, using google scholar as a keyword does not seem to fit what is discussed in the paper.

**Statement of the problem**: It identifies the lack of studies that cumulatively explain the evolution of PCK from its inception. However, the authors would consider presenting the arguments logically instead of mixing up PCK and TPACK in a zigzag way by focusing their attention first on PCK when done then TPACK.

**Research questions:** Although the title and various places it is claimed that the study focuses on the learning of mathematics, none of the research questions is focused on mathematics.

**Data selection**: Selection of articles is well articulated. However, the authors have not explained why they chose google scholar as the sole database for their search. They may also explain why they ended on pages 10 and 30 in search of review articles and empirical studies respectively.

**Nature of selected articles:** The author concluded that “therefore, our analyses only considered journal articles” without explaining why so. Were other types of sources not relevant/fit for the study? They may consider explaining why they selected only journal articles.

**Writing style:** The authors have written several sentences that begin with numerals. They need to rephrase them and ensure no sentence starts with a numeral. They also use one-sentence paragraphs, a style uncommon in academic writing.

**Data analysis: **This section is supposed to explain how the results presented in the following sections were obtained. However, the authors seem to give more space to what the articles were about – an explanation that fits in their preceding section named “extended sources and nature of selected articles”. For example, all the tables and figures in this section are about the articles selected (when they were published, where they were published, and their subjects) and not about how the selected articles were analyzed. Only the first paragraph of the section talks about how findings were presented but even this is not preceded by how such presented finding were found through analysis. The authors may consider focusing their attention on explaining how they went about analyzing the documents they selected.

**Results and discussion:** The section seems to be divided into two major subsections namely PCK framework and PCK interventions. Under each subsection, there are presented lists of studies that the authors discuss one after another. The studies are discussed as isolated ideas that do not inform or complement each other. There is no explanation why the second study or the third study etc. are important in/or add to the idea presented in the preceding studies. Also, there is no synthesis as to what we cannot learn from the listed studies. What is (not) common about them? What do we now learn about PCK and TPACK from them? How about mathematics? What have the studies shown in general about the learning of mathematics? Is PCK or TPACK sufficient in explaining effective learning of mathematics? What component of the frameworks should/has not been well addressed by teachers for effective learning of mathematics? Also, the studies are presented separately from each other, each having its own heading, where some are discussed using one sentence paragraph. The authors may consider explaining in a logical flow how PCK for example, is understood from some two to three papers and how this understanding is corroborated/advanced/contradicted/etc. by other studies. They may do so by connecting the ideas from the studies to one another and explicitly showing what we can now learn from the cumulative trend.

**Conclusion:** Although the conclusion hits the point, especially in the last paragraph, the preceding paragraphs seem to supplement what may have been missed in the discussion. As such much of its content does not sound as concluding.

Is the work clearly and accurately presented and does it cite the current literature?

Partly

If applicable, is the statistical analysis and its interpretation appropriate?

Not applicable

Are all the source data underlying the results available to ensure full reproducibility?

Yes

Is the study design appropriate and is the work technically sound?

Yes

Are the conclusions drawn adequately supported by the results?

Partly

Are sufficient details of methods and analysis provided to allow replication by others?

Partly

Reviewer Expertise:

Mathematics education, teacher education, and technology integration in education.

I confirm that I have read this submission and believe that I have an appropriate level of expertise to confirm that it is of an acceptable scientific standard, however I have significant reservations, as outlined above.

# Response

**Published:** 2023-08-11

**Authors:** Hashituky Telesphore Habiyaremye

**Affiliations:** University of Rwanda, Rwanda

Reviewer comments:

 Summary: The study is a literature review. It is designed to discuss the trend in PCK and TPACK studies and how they influence effective learning, especially in mathematics. The study reviews different literature in the area to respond to two research questions. Data sources are obtained from the Google Scholar database and screened for relevance. Data analysis is done to uncover the trends in PCK and TPACK and their effectiveness in explaining learning. As such findings are presented first by listing and explaining studies that explain the PCK or TPACK framework and those that explain the use of the framework in explaining learning especially in mathematics. The topic is current and may inform future studies on the evolution and debate in PCK and TPACK and how they have been used to influence learning. Specific comments about possible areas for improvement of the paper are provided as follows:

**Author response: Thank you so much for appreciating our study contribution! We have framed our study based on your suggestions.**

 Keywords: although the authors used the Google Scholar database as the source of their articles, using google scholar as a keyword does not seem to fit what is discussed in the paper.

**Author response: We deleted the google scholar keyword.**

 Statement of the problem: It identifies the lack of studies that cumulatively explain the evolution of PCK from its inception. However, the authors would consider presenting the arguments logically instead of mixing up PCK and TPACK in a zigzag way by focusing their attention first on PCK when done then TPACK.

**Author response: The problem statement was rewritten by focusing attention first on PCK and then TPACK.**

 Research questions: Although the title and various places it is claimed that the study focuses on the learning of mathematics, none of the research questions is focused on mathematics.

 Author response: The problem statement was rewritten by focusing attention first on PCK and then TPACK.

**Author response: “in teaching and learning mathematics” was added.**

 Data selection: Selection of articles is well articulated. However, the authors have not explained why they chose google scholar as the sole database for their search. They may also explain why they ended on pages 10 and 30 in search of review articles and empirical studies respectively.

**Author response: The choice of Google Scholar was influenced by its extensive coverage of scholarly articles and user-friendly interface, facilitating efficient searches to access a wide array of academic publications. The search ended on page 10 in search of review articles because the first seven pages exhausted all articles written in review form, such as a review of literature, systematic review, meta-analysis, etc. This means that we checked pages eight to 10, had no results, and stopped checking after page 10. Eventually, the search for empirical studies ended on page 30 because no mathematics-related paper was available.**

 Nature of selected articles: The author concluded that “therefore, our analyses only considered journal articles” without explaining why so. Were other types of sources not relevant/fit for the study? They may consider explaining why they selected only journal articles.

**Author response: We considered journal articles due to their rigorous peer-review process that ensures higher academic scrutiny and quality.**

 Writing style: The authors have written several sentences that begin with numerals. They need to rephrase them and ensure no sentence starts with a numeral. They also use one-sentence paragraphs, a style uncommon in academic writing.

**Author response: The journal editor suggested beginning with numerals in the pre-assessment review round.**

 Data analysis: This section is supposed to explain how the results presented in the following sections were obtained. However, the authors seem to give more space to what the articles were about – an explanation that fits in their preceding section named “extended sources and nature of selected articles”. For example, all the tables and figures in this section are about the articles selected (when they were published, where they were published, and their subjects) and not about how the selected articles were analyzed. Only the first paragraph of the section talks about how findings were presented but even this is not preceded by how such presented finding were found through analysis. The authors may consider focusing their attention on explaining how they went about analyzing the documents they selected.

**Author response: This section refocused on explaining how we analyzed the documents we selected. We then moved other texts under the previous subsection of extended sources and the nature of selected articles. The following was added: “To reveal trends of the PCK framework, we identified the year of publication, author of the study, model of PCK, and construct (whether PCK or TPACK) for each study. To reveal the effectiveness of PCK and TPACK interventions, we identified the construct, theoretical framework used to frame the study, research design, teaching intervention, topic, and analysis method for each study.” Figure 1 was changed to Figure 4 and maintained in this section, showing data analysis software usage.**

 Results and discussion: The section seems to be divided into two major subsections namely PCK framework and PCK interventions. Under each subsection are presented lists of studies that the authors discuss one after another. The studies are discussed as isolated ideas that do not inform or complement each other. There is no explanation why the second study or the thirdstudy etc. are important in/or add to the idea presented in the preceding studies. Also, there is no synthesis as to what we cannot learn from the listed studies. What is (not) common about them? What do we now learn about PCK and TPACK from them? How about mathematics? What have the studies shown in general about the learning of mathematics? Is PCK or TPACK sufficient in explaining effective learning of mathematics? What component of the frameworks should/has not been well addressed by teachers for effective learning of mathematics? Also, the studies are presented separately from each other, each having its own heading, where some are discussed using one sentence paragraph. The authors may consider explaining in a logical flow how PCK for example, is understood from some two to three papers and how this understanding is corroborated/advanced/contradicted/etc. by other studies. They may do so by connecting the ideas from the studies to one another and explicitly showing what we can now learn from the cumulative trend.

**Author response: Each article's discussion in more detail was changed to a more thorough connection, and discussion together to provide compromise, same trends, opposing trends, and contradiction from one to the other.**

 Conclusion: Although the conclusion hits the point, especially in the last paragraph, the preceding paragraphs seem to supplement what may have been missed in the discussion. As such much of its content does not sound as concluding.

**Author response: The whole conclusion was revised.**
